# Field study of parasitic contamination of fruits, vegetables and leafy greens in the Ecuadorian Andes

**DOI:** 10.12688/f1000research.132957.1

**Published:** 2023-05-23

**Authors:** Luisa Carolina González-Ramírez, Pablo Djabayan-Djibeyan, José G. Prato, Cecilia Alejandra García Ríos, Julio César Carrero, María Trelis, Màrius Vicent Fuentes

**Affiliations:** 1 Grupo de Investigación "Análisis de Muestras Biológicas y Forenses", Carrera de Laboratorio Clínico, Facultad de Ciencias de la Salud, Universidad Nacional de Chimborazo, Riobamba, Chimborazo Province, 060103, Ecuador; 2 Grupo de Investigación "Salud Pública", Carrera de Medicina, Facultad de Ciencias de la Salud, Universidad Nacional de Chimborazo, Riobamba, Chimborazo Province, 060103, Ecuador; 3 Grupo de Investigación “Estudios Interdisciplinarios”, Ingeniería Ambiental, Facultad de Ingeniería, Universidad Nacional de Chimborazo, Riobamba, Chimborazo Province, 060103, Ecuador; 4 Facultad de Salud Pública, Escuela Superior Politecnica de Chimborazo, Riobamba, Chimborazo Province, 060103, Ecuador; 5 Departamento de Inmunología, Instituto de Investigaciones Biomédicas, Universidad Nacional Autónoma de México, Mexico City, Mexico City, 04510, Mexico; 6 Research Group "Parasites and Health", Departament de Farmàcia i Tecnologia Farmacèutica i Parasitologia, Facultat de Farmàcia, Universitat de València, Burjassot/Valencia, Comunidad Valenciana, 46010, Spain; 7 Joint Research Unit on Endocrinology, Nutrition and Clinical Dietetics, Universitat de València - Health Research Institute La Fe (IISLAFE), Valencia, Valencian Community, 46026, Spain

**Keywords:** agricultural production, food, transmission, parasites, fruits, vegetables, leafy greens

## Abstract

**Background**: Raw vegetables have been considered vehicles of enteroparasites. South American countries are among the most important exporters of fresh vegetables; Ecuador has tropical climates and soils rich in organic matter that allow it to harvest throughout the year for sale to different countries. The aim of the study was to assess the occurrence of the parasitic contamination of fruits, vegetables and leafy greens grown in an agricultural area of the Ecuadorian Andes.

**Methods:** A field study, cross-sectional, snowball sampling was conducted on 1,416 samples (516 fruits, 488 vegetables, and 412 leafy greens). Each sample were washed with water, and the resulting solution after removing the vegetables, was subjected to 24-hour sedimentation. The concentrated sediment underwent microscopic analysis.

**Results**: Parasites were detected in 63.4% of the samples, leafy greens were the most contaminated (76.9%) (P<0.0001), (vegetables 67.8% and fruit 48.4%), of these, cabbage (100%), onions (84%) and strawberries (60.2%) were the most contaminated. Protozoa were more frequent (49.6%) than helminths (15.5%) (P<0.0001). *Blastocystis* sp. (33.5%) was the highest, followed by *Eimeria* spp. (26.3%), *Entamoeba* spp. (10.3%), *Giardia* spp. (8.3%), *Balantidium* spp. (6.9%); *Cryptosporidium* spp. (6.6%), *Cyclospora* spp. (4.4%), *Cystoisospora* spp. (0.5%); Strongylida (15.5%) and *Ascaris* spp. (0.4%).

**Conclusion**: The consumption of fruits, vegetables, and leafy greens from these crops is a possible source of infection to humans and animals in this area or in nonendemic areas where these products are marketed. This study establishes the need for strict hygienic measures in growing; this will be properly achieved by the treatment of the soil, manure and water used for cultivation.

## Introduction

The consumption of fruits and vegetables provides essential nutrients for a healthy diet; however, raw vegetables are among the main vehicles for enteroparasites ( FAO/WHO, 2008; Punsawad *et al*., 2019; Al Nahhas & Aboualchamat, 2020), and contamination can occur at any stage of the food production and marketing chain ( Trelis *et al*., 2022). Various studies have focused on cultivation and harvesting stages as a risk phase, due to inadequate farming hygiene, contact with soil contaminated with human and animal feces (by direct defecation on crops or their fertilization), and the use of contaminated water for irrigation, dilution of pesticides, or washing equipment ( Pérez-Cordón *et al*., 2008; Efstratiou *et al*., 2017; Ercumen *et al*., 2017; Luz *et al*., 2017; Karshima, 2018; Trelis *et al*., 2022).

Although parasites do not multiply in food, they can survive for months, and resistance to some chemical and physical inactivating agents aggravates the situation ( Ramos *et al*., 2013), so parasites can infect individuals in areas far away from the production site ( Dixon *et al*., 2013; Dixon, 2016; Caradonna *et al*., 2017; Machado-Moreira *et al*., 2019; Li *et al*., 2020).

South American countries are among the most important exporters of fresh vegetables. Ecuador has a tropical climate and soils rich in organic matter that allow it to harvest fruits, vegetables, and grains throughout the year for sale to different countries. According to figures from the Agriculture and Livestock Ministry, during the period of 2014-2018, Ecuador raised more than $3,500 million by exporting 6,000,000 tons of fruits and vegetables, including 212 tons of bananas, 139 tons of baby bananas, 81 tons of pineapples, 74 tons of broccoli and 60 tons of mangoes ( Ministerio de Agricultura y Ganadería Ecuador, 2020).

Imports of fresh produce from endemic countries have contributed to the spread of parasites to nonendemic countries; diarrhoea epidemics have been reported from the consumption of raspberries, tomatoes, peppers, onions, carrots and radishes (FAO/WHO, 2008; Dixon, 2016; Machado-Moreira *et al*., 2019; Li *et al*., 2020). The WHO highlights the importance of leafy greens (spinach, lettuce, cabbage, watercress, basil, mint, coriander and parsley) as vehicles for food-borne parasites ( FAO/WHO, 2008), among which are *Cyclospora cayetanensis, Cryptosporidium* spp. and *Giardia duodenalis, Toxoplasma gondii*, *Entamoeba histolytica*, *Blastocystis* sp., *Cystoisospora belli*, *Balantidium coli*, *Dientamoeba fragilis* and geohelminths ( Ramos *et al*., 2013; Gamble, 2015; Dixon, 2016; Caradonna *et al*., 2017; Karshima, 2018; Robertson, 2018; Trelis *et al*., 2022).

In Ecuador, there is a dramatic health situation that affects all rural Andean regions, especially those located at high altitudes, which are mostly inhabited by indigenous populations that have agriculture, livestock, and other animal husbandry as a means of subsistence ( González-Ramírez *et al*., 2021, 2022). Moreover, it is important to note that farmers need to learn good agricultural practices since they may not have adequate training, confidence, or economic resources to maintain crops and animals with proper hygiene to guarantee food quality production.

A review indicated that there are no studies carried out in other provinces of Ecuador; only one report evaluated the risk factors associated with parasite transmission and described a total of 70.6% contamination of fruits and vegetables grown in six rural communities in the parish of San Andres, Chimborazo province ( González-Ramírez *et al*., 2022). Due to this alarming contamination figure, we proposed evaluating the parasitic contamination of all products (fruits, vegetables and leafy greens) grown in the capital of San Andres, an agricultural zone of the Ecuadorian Andes. The probable causes of produce contamination during the primary production phases are likely due to sanitary control during these stages of the production chain; these need to be analyzed to minimize the risk of infections, which will benefit exports with favourable consequences on the country’s economy.

## Methods

### Study area

The study area was the community of San Andrés, Guano canton, Chimborazo province of Ecuador, located at 3,900 meters above sea level. The local temperature ranges between 5-18 °C, and rainfall varies between 500-1,000 mm/year. There are two rainy periods, February to May and October to November; the remaining months are transitional with moderate rains. Evapotranspiration affects the drought of the soil, which originates from volcanic ashes of variable textures, most of which are shallow silty loam, with a pH of 4.5 to 6.5. There are loamy soils in the areas with the highest agricultural production, but they are affected by chemical fertilizers. There are also sandy soils with low fertility because they do not retain moisture and nutrients; the latter and the action of steep slopes make them susceptible to erosive processes; consequently, crops and sowing grass are not abundant. However, agricultural activity is 34.5%, and cattle breeding activity is 50.4%; these two are the main means of financial income for the local population ( PDOT San Andrés, 2015).

Government records indicate that 47.9% of the rural population of Ecuador lives in poverty, with an average monthly family income of $84.05, and 27.5% living in extreme poverty, with an average income of $47.70. The province of Chimborazo has an illiteracy rate of 13.5%, and the community of San Andrés has an indigenous population of 36.9% ( INEC, 2020). Hence, their training is based on habits and customs acquired from their ancestors, which may contribute to as a lack of basic hygiene and sanitary measures. The most remote communities have built septic tanks, and the communities closest to the capital have sewers; however, both drain wastewater into rivers and streams ( PDOT San Andrés, 2015).

### Investigation design

A field study, cross-sectional, was carried out between May and December 2019 (1 month of rain/7 months of drought). The snowball sampling technique was applied, whereby a grower helped locate the nearest farm and so on. All types of products found were included in the sampling (1,416 samples in total); the inclusion criteria were that all agricultural products must come from San Andrés fields and those not cultivated in the community were excluded.

### Sampling

The total of 1,416 samples analyzed included 516 fruits of 8 types: *Fragaria ananassa* (strawberry), *Rubus glaucus* (blackberry), *Physalis peruviana* (uvilla), *Prunus persica* (peach), *Citrus limon* (lemon), *Psidium guajava* (guava), *Ficus carica* (fig), and *Solanum lycopersicum* (tomato); 488 vegetables of 9 types: *Allium cepa* var. rosum (red onions) and *Allium cepa L* (white onions), *Solanum tuberosum* (potato), *Daucus carota* (carrot), *Raphanus sativus* (radish), *Beta vulgaris* (beet), *Capsicum annuum* (sweet pepper), *Capsicum frutescens* (chili pepper), and *Lupinus mutabilis* (bean chochos) and 412 leafy greens of 8 types: *Medicago sativa* (alfalfa), *Lactuca sativa* (lettuce), *Brassica oleracea* (cabbage), *Beta vulgaris* (chard), *Petroselinum crispum* (parsley), *Coriandrum sativum* (cilantro), *Apium graveolens* (celery), and *Nasturtium officinale* (watercress).

All samples were obtained from the owners' fields and stored in hermetically sealed propylene bags. Each sample was labelled indicating the plant species name, origin, date, and time of collection. The samples were immediately transported in their containers with cooling gels to the Research Laboratory of the Faculty of Health Sciences, National University of Chimborazo, to be processed within one hour of collection.

### Ethical considerations

The sampling was carried out with the appropriate permission of the Cantonal and Parochial Decentralized Autonomous Governments. All farmers collected samples of their own crops (as they always do), knowing that the study benefits the community, without compromising the health of the population with respect to bioethical principles.

### Parasitological analysis

The processing protocol for the parasitological analysis of all samples, previously described by Rivero de Rodríguez *et al*. (1998), was utilized. For the processing of the samples, 75 g of vegetables, fruits or green leaves were taken and added to 500 mL of previously filtered and boiled water. The contents were stirred with the help of a magnetic stirrer for 1 hour, the remains of the vegetable were removed and the solution was left to stand for 24 hours. Subsequently, the solution was decanted into a separatory funnel and the first fraction was collected in 15 mL tubes to be subjected to centrifugation for 5 min at 800 xg. Once the concentrate or sediment was separated, the supernatant was discarded and the precipitate was reconstituted in 400 μL of saline (0.85%). Each sample was observed under a light microscope (Nikon E200) using 10x and 40x objectives. In addition, iodized solution and the ocular micrometer were used when necessary, for stain parasitic structures or to measure the dimensions for their recognition. Additionally, a smear was made with one drop from the pellet and prepared for acid-fast staining (using a modified Zielh-Neelsen technique) for coccidia oocyst detection and identification after measurement, mainly *Crytosporidium* and *Cyclospora*, and subsequent microscopic assessment (100×) ( García *et al*., 1983).

### Statistical analysis

The database made in Microsoft Excel was exported to SPSS Statistic 26.0 software (IBM, New York, NY, USA). The difference in parasitic contamination between the various categories of plant products and the predominant parasite type in each plant species were compared using Pearson's chi-square test (χ ^2^) and Fisher's exact test, when appropriate. A *P* value <0.05 was considered statistically significant.

## Results

When analyzing the different crop products, a total of 898 (63.4%) were contaminated by parasites. A statistically significant difference between the overall contamination rates, the leafy greens (76.9%) were more contaminated than vegetables (67.8%) and fruits (48.4%) ( *P*<0.0001). Also identified were 15 protozoa and 2 helminth nematodes, protozoa also had a higher prevalence (49.6%) than nematodes (15.5%) ( *P*<0.0001). *Blastocystis* sp. was outstanding among protozoa (33.5%) ( *P*<0.0001), followed by *Eimeria* spp. (26.3%), *Entamoeba* spp. (10.3%), *Giardia* spp. (8.3%) and *Cryptosporidium* spp. (6.6%). Between the nematodes, Strongylida were more frequent than *Ascaris* spp. ( *P*<0.0001) (see Table 1).

**Table 1.** Distribution of parasites according to the plant type analysed.

Parasites	Fruits			Vegetables			Leafy Greens			Total		
	n=516	%	IC	n=488	%	IC	n=412	%	IC	n=1416	%	IC
*Blastocystis* sp.	193	37.4	(33.2-41.6)	134	27.5	(23.5-31.4)	148	35.9	(31.3-40.6)	475	33.5	(31.1-36)
*Entamoeba* spp.	29	5.6	(3.6-7.6)	48	9.8	(7.2-12.5)	69	16.7	(13.1-20.4)	146	10.3	(8.7-11.9)
*E. coli*	9	1.7	(0.6-2.9)	11	2.3	(0.9-3.6)	3	0.7	(0-1.6)	23	1.6	(1-2.3)
*E. hartmanni*	2	0.4	(0-0.9)	2	0.4	(0-1)	1	0.2	(0-0.7)	5	0.4	(0-0.7)
*Endolimax nana*	31	6	(4.0-8.1)	9	1.8	(0.7-3.0)	15	3.6	(1.8-5.5)	55	3.9	(2.9-4.9)
*Iodamoeba buetschlii*	2	0.4	(0-0.9)	2	0.4	(0-1)	1	0.2	(0-0.7)	5	0.4	(0-0.7)
*Giardia* spp.	26	5	(3.2-7)	40	8.2	(5.8-10.6)	52	12.6	(9.4-15.8)	118	8.3	(6.9-9.8)
*Chilomastix* spp.	6	1.2	(0.3-2.1)	8	1.6	(0.5-2.8)	3	0.7	(0-1.6)	17	1.2	(0.6-1.8)
*Retortamonas* spp.	0	0	0	1	0.2	(0-0.6)	0	0	0	1	0.1	(0-0.2)
*Enteromonas* spp.	3	0.6	(0-1.3)	0	0	0	1	0.2	(0-0.7)	4	0.3	(0-0.6)
*Cryptosporidium* spp.	39	7.6	(5.3-9.9)	30	6.1	(4.0-8.3)	24	5.8	(3.6-8.1)	93	6.6	(5.3-7.9)
*Cyclospora* spp.	31	6	(4.0-8.1)	20	4.1	(2.3-5.9)	12	2.9	(1.3-4.5)	63	4.4	(3.4-5.5)
*Cystoisospora* spp.	3	0.6	(0-1.2)	2	0.4	(0-1)	2	0.5	(0-1.2)	7	0.5	(0.1-0.9)
*Eimeria* spp.	112	21.7	(18.1-25.3)	123	25.2	(21.4-29.1)	138	33.5	(28.9-38.1)	373	26.3	(24.0-28.6)
*Balantidium* spp.	5	1	(0.1-1.8)	30	6.1	(4.0-8.3)	62	15	(11.6-18.5)	97	6.9	(5.5-8.2)
Protozoa	220	42.6	(38.4-46.9)	230	47.1	(42.7-51.6)	253	61.4	(56.7-66.1)	703	49.6	(47-52.3)
*Ascaris* spp.	0	0	0	3	0.6	(0-1.3)	2	0.5	(0-1.2)	5	0.4	(0-0.7)
Strongylida	32	6.2	(4.1-8.3)	115	23.6	(20.4-28.0)	70	17	(13.4-20.6)	217	15.3	(13.7-17.4)
Helminths	32	6.2	(4.1-8.3)	118	24.2	(20.4-28.0)	70	17	(13.4-20.6)	220	15.5	(13.7-17.4)
Total	250	48.4	(44.1-52.7)	331	67.8	(63.7-71.9)	317	76.9	(72.8-81)	898	63.4	(60.9-65.9)

When comparing the percentages of contamination of fruits, vegetables and leafy greens by the different parasites, the statistical analysis determined that, in fruits, a higher prevalence of *Blastocystis* (37.4%) ( *P*=0.0018), *Cryptosporidium* (7.6%) ( *P*<0.0001), *Cyclospora* (6%) ( *P*<0.0001) and *Endolimax nana* (6%) ( *P*=0.0028) was found. Vegetables were mostly contaminated by helminths (24.2%) ( *P*<0.0001), represented mainly Strongylida (23.6%) ( *P*<0.0001). Finally, the leafy greens showed greater contamination by *Eimeria* (33.5%) ( *P*=0.0002), *Entamoeba* spp. (16.7%) ( *P*<0.0001), *Balantidium* (15.0%) ( *P*<0.0001) and *Giardia* (12.6%) ( *P*=0.0002), which comprised the highest contamination with protozoa (61.4%) ( *P*<0.0001) and a total parasitic contamination of 76.9% ( *P*<0.0001) (see Table 1).

Table 2 summarizes the results obtained according to the type of fruit, the highest number of protozoa was found in strawberries (60.2%) ( *P*<0.0001), with *Blastocystis* sp. (59.2%) ( *P*<0.0001), *E. nana* (17.35%) ( *P*<0.0001) and *Cyclospora* spp. (14.3%) ( *P*=0.0011) in contrast to peaches, which were more often contaminated with helminths (30%) ( *P*<0.0001).

**Table 2.** Distribution of parasites according to the fruit type analysed.

Parasites	Strawberry		Blackberry		Uvilla		Peach		Lemon		Guava		Fig		Tomato		Total	
	n=98	%	n=83	%	n=56	%	n=50	%	n=56	%	n=57	%	n=50	%	n=66	%	n=516	%
*Blastocystis* sp.	58	59.2	35	42.2	19	33.9	24	48	14	25	19	33.3	15	30	9	13.6	193	37.4
*Entamoeba* spp.	4	4.1	5	6	0	0	2	4	0	0	9	15.8	3	6	6	9.1	29	5.6
*E. coli*	4	4.1	4	4.8	0	0	0	0	0	0	1	1.8	0	0	0	0	9	1.7
*E. hartmanni*	0	0	1	1.2	0	0	0	0	0	0	0	0	0	0	1	1.5	2	0.4
*Endolimax nana*	17	17.3	6	7.2	0	0	3	6	1	1.8	3	5.3	0	0	1	1.5	31	6
*Iodamoeba buetschlii*	0	0	0	0	0	0	0	0	0	0	0	0	0	0	2	3	2	0.4
*Giardia* spp.	10	10.2	4	4.8	1	1.8	1	2	1	1.8	1	1.8	2	4	6	9.1	26	5
*Chilomastix* spp.	0	0	3	3.6	0	0	0	0	0	0	0	0	2	4	1	1.5	6	1.2
*Retortamonas* spp.	3	3.1	0	0	0	0	0	0	0	0	0	0	0	0	0	0	3	0.6
*Enteromonas* spp.	0	0	0	0	0	0	0	0	0	0	0	0	0	0	0	0	0	0
*Cryptosporidium* spp.	11	11.2	11	13.3	4	7.1	4	8	1	1.8	4	7	1	2	3	4.5	39	7.6
*Cyclospora* spp.	14	14.3	7	8.4	5	8.9	0	0	1	1.8	3	5.3	0	0	1	1.5	31	6
*Cystoisospora* spp.	1	1	1	1.2	1	1.8	0	0	0	0	0	0	0	0	0	0	3	0.6
*Eimeria* spp.	20	20.4	23	27.7	24	42.9	10	20	1	1.8	5	8.8	17	34	12	18.2	112	21.7
*Balantidium* spp.	3	3.1	0	0	1	1.8	0	0	0	0	0	0	0	0	1	1.5	5	1
Protozoa	59	60.2	46	55.4	24	42.9	16	32	3	5.4	20	35.1	23	46	29	43.9	220	42.6
*Ascaris* spp.	0	0	0	0	0	0	0	0	0	0	0	0	0	0	0	0	0	0
Strongylida	0	0	0	0	9	16.1	15	30	5	8.9	0	0	1	2	2	3	32	6.2
Helminths	0	0	0	0	9	16.1	15	30	5	8.9	0	0	1	2	2	3	32	6.2
Total	59	60.2	46	55.4	32	57.1	31	62	7	12.5	20	35.1	24	48	31	47	250	48.4

Parasitic contamination in the different types of vegetables is detailed in Table 3, the highest was found in red (84%) and white (82.4%) onions, followed by chili pepper (78%) ( *P*<0.0001). It is important to highlight the level of contamination detected in other vegetables that are eaten raw (carrot 66%, radish 72.1% and pepper 44%). In the analysis to contrast the total parasitic contamination between protozoa (47.1%) and helminths (24.2%), it was possible to verify a higher frequency of protozoa ( *P*<0.0001).

**Table 3.** Distribution of parasites according to the vegetable type analysed.

Parasites	Red onion		White onion		Potato		Carrot		Radish		Beet		Pepper		Chili pepper		Beans chocho		Total	
	n=50	%	n=51	%	n=52	%	n=53	%	n=61	%	n=51	%	n=50	%	n=50	%	n=70	%	n=488	%
*Blastocystis* sp.	28	56	16	31.4	11	21.2	12	22.6	22	36.1	9	17.6	6	12	14	28	16	22.9	134	27.5
*Entamoeba* spp.	3	6	1	2	6	11.5	6	11.3	8	13.1	13	25.5	1	2	7	14	3	4.3	48	9.8
*E. coli*	0	0	2	3.9	3	5.8	1	1.9	5	8.2	0	0.0	0	0	0	0	0	0	11	2.3
*E. hartmanni*	0	0	1	2	1	1.9	0	0	0	0	0	0.0	0	0	0	0	0	0	2	0.4
*Endolimax nana*	0	0	4	7.8	1	1.9	2	3.8	0	0	0	0.0	0	0	2	4	0	0	9	1.8
*Iodamoeba buetschlii*	0	0	0	0	0	0	1	1.9	0	0	0	0.0	1	2	0	0	0	0	2	0.4
*Giardia* spp.	8	16	6	11.8	1	1.9	3	5.7	3	4.9	2	3.9	7	14	7	14	3	4.3	40	8.2
*Chilomastix* spp.	4	8	2	3.9	1	1.9	0	0	0	0	0	0	0	0	1	2	0	0	8	1.6
*Retortamonas* spp.	0	0	0	0	0	0	1	1.9	0	0	0	0	0	0	0	0	0	0	1	0.2
*Enteromonas* spp.	0	0	0	0	0	0	0	0	0	0	0	0	0	0	0	0	0	0	0	0
*Cryptosporidium* spp.	3	6	7	13.7	2	3.8	6	11.3	3	4.9	2	3.9	0	0	2	4	5	7.1	30	6.1
*Cyclospora* spp.	2	4	2	3.9	1	1.9	5	9.4	3	4.9	6	11.8	0	0	0	0	1	1.4	20	4.1
*Cystoisospora* spp.	0	0	0	0	1	1.9	1	1.9	0	0	0	0	0	0	0	0	0	0	2	0.4
*Eimeria* spp.	10	20	10	19.6	15	28.8	13	24.5	22	36.1	17	33.3	12	24	17	34	7	10	123	25.2
*Balantidium* spp.	1	2	3	5.9	3	5.8	4	7.5	7	11.5	6	11.8	1	2	3	6	2	2.9	30	6.1
Protozoa	24	48	28	54.9	27	51.9	25	47.2	33	54.1	28	54.9	19	38	27	54	19	27.1	230	47.1
*Ascaris* spp.	0	0	0	0	0	0	0	0	0	0	2	3.9	1	2	0	0	0	0	3	0.6
Strongylida	16	32	28	54.9	22	42.3	7	13.2	15	24.6	11	21.6	1	2	15	30	0	0	115	23.6
Helminths	16	32	28	54.9	22	42.3	7	13.2	15	24.6	13	25.5	2	4	15	30	0	0	118	24.2
Total	42	84	42	82.4	40	76.9	35	66.0	44	72.1	37	72.5	22	44	39	78	30	42.9	331	67.8

The parasitic contamination in leafy greens was significantly different among types ( Table 4); greater percentages of parasites were found in cabbage (100%), alfalfa (90.2%) and parsley (82.4%). It was possible to verify higher contamination of the cabbage with *Eimeria* (53.8%) ( *P*<0.0001) and with *Endolimax nana* (13.5%) ( *P*=0.0002), lettuce with *Entamoeba* spp. (36.2%) ( *P*<0.0001), and parsley with *Blastocystis* (56.9%) ( *P*=0.0071).

**Table 4.** Distribution of parasites according to the leafy green type analysed.

Parasites	Alfalfa		Lettuce		Cabbage		Chard		Parsley		Coriander		Celery		Watercress		Total	
	n=51	%	n=58	%	n=52	%	n=50	%	n=51	%	n=50	%	n=50	%	n=50	%	n=412	%
*Blastocystis* sp.	13	25.5	24	41.4	20	38.5	12	24	29	56.9	19	38	12	24	19	37.3	148	35.9
*Entamoeba* spp.	4	7.8	21	36.2	16	30.8	3	6	2	3.9	6	12	7	14	10	19.6	69	16.7
*E. coli*	0	0	1	1.7	1	1.9	0	0	1	2	0	0	0	0	0	0	3	0.7
*E. hartmanni*	0	0	0	0	0	0	0	0	0	0	0	0	1	2	0	0	1	0.2
*Endolimax nana*	5	9.8	0	0	7	13.5	0	0	2	3.9	1	2	0	0	0	0	15	3.6
*Iodamoeba buetschlii*	0	0	0	0	1	1.9	0	0	0	0	0	0	0	0	0	0	1	0.2
*Giardia* spp.	5	9.8	5	8.6	9	17.3	4	8	7	13.7	8	16	4	8	10	19.6	52	12.6
*Chilomastix* spp.	0	0	0	0	0	0	1	2	2	3.9	0	0	0	0	0	0	3	0.7
*Retortamonas* spp.	0	0	0	0	0	0	0	0	0	0	0	0	0	0	0	0	0	0
*Enteromonas* spp.	0	0	0	0	1	1.9	0	0	0	0	0	0	0	0	0	0	1	0.2
*Cryptosporidium* spp.	1	2	3	5.2	2	3.8	1	2	5	9.8	3	6	4	8	5	9.8	24	5.8
*Cyclospora* spp.	0	0	1	1.7	4	7.7	0	0	1	2	2	4	4	8	0	0	12	2.9
*Cystoisospora* spp.	0	0	0	0	1	1.9	1	2	0	0	0	0	0	0	0	0	2	0.5
*Eimeria* spp.	26	51	17	29.3	28	53.8	14	28	13	25.5	9	18	17	34	14	27.5	138	33.5
*Balantidium* spp.	7	13.7	8	13.8	11	21.2	5	10	5	9.8	8	16	9	18	9	17.6	62	15
Protozoa	33	64.7	37	63.8	45	86.5	23	46	33	64.7	25	50	30	60	27	52.9	253	61.4
*Ascaris* spp.	1	2	0	0	0	0	0	0	0	0	0	0	1	2	0	0	2	0.5
Strongylida	13	25.5	11	19	11	21.2	6	12	8	15.7	6	12	9	18	6	12.0	70	17
Helminths	13	25.5	11	19	11	21.2	6	12	8	15.7	6	12	9	18	6	12.0	70	17
Total	46	90.2	43	74.1	52	100	30	60	42	82.4	31	62	39	78	34	66.7	317	76.9

Comparative analysis of parasitic contamination rates detected between fruits and vegetables/leafy greens ( Table 5) showed higher parasites percentages in vegetables/leafy greens, with significant differences in the total (72%) ( *P*<0.0001), protozoa (53.7%) ( *P*<0.0001) and helminths (20.9%) ( *P*<0.0001). A higher prevalence of *Eimeria* (29%) ( *P*=0.0027), *Entamoeba* spp. (13%) ( *P*<0.0001), *Giardia* (10.2%) ( *P*=0.0007), and *Balantidium* (10.2%) ( *P*<0.0001) were found. In contrast, higher percentages of *Blastocystis* (37.4%) ( *P*=0.0199) and *Cyclospora* (6%) ( *P*=0.0313) were found in fruits.

**Table 5.** Comparation of parasitic contamination between fruits, vegetables and leafy greens.

Parasites	Fruit			Vegetables + Greens leafy		
	n=516	%	IC	n=900	%	IC
*Blastocystis* sp.	193	37.4	(33.2-41.6)	282	31.3	(28.3-34.3)
*Entamoeba* spp.	29	5.6	(3.6-7.6)	117	13	(10.8-15.2)
*E. coli*	9	1.7	(0.6-2.9)	14	1.6	(0.8-2.4)
*E. hartmanni*	2	0.4	(0-0.9)	3	0.3	(0-0.7)
*Endolimax nana*	31	6	(4.0-8.1)	24	2.7	(1.6-3.8)
*Iodamoeba buetschlii*	2	0.4	(0-0.9)	3	0.3	(0-0.7)
*Giardia* spp.	26	5	(3.2-7.0)	92	10.2	(8.2-12.2)
*Chilomastix* spp.	6	1.2	(0.3-2.1)	11	1.2	(0.5-1.9)
*Retortamonas* spp.	0	0	0	1	0.1	(0-0.3)
*Enteromonas* spp.	3	0.6	(0-1.3)	1	0.1	(0-0.3)
*Cryptosporidium* spp.	39	7.6	(5.3-9.9)	54	6	(4.4-7.6)
*Cyclospora* spp.	31	6	(4.0-8.1)	32	3.6	(2.4-4.8)
*Cystoisospora* spp.	3	0.6	(0-1.2)	4	0.4	(0-.80)
*Eimeria* spp.	112	21.7	(18.1-25.3)	261	29	(26.0-32.0)
*Balantidium* spp.	5	1	(0.1-1.8)	92	10.2	(8.2-12.2)
Protozoa	220	42.6	(38.4-46.9)	483	53.7	(50.4-57.0)
*Ascaris* spp.	0	0	0	5	0.6	(0.1-1.1)
Strongylida	32	6.2	(4.1-8.3)	185	20.6	(18.2-23.6)
Helminths	32	6.2	(4.1-8.3)	188	20.9	(18.2-23.6)
Total	250	48.4	(44.1-52.7)	648	72	(69.1-74.9)

When parasitic contamination was compared between leafy greens (76.9%) and vegetables (67.8%), a statistically significant difference was found ( *P* =0.0024) (see Table 6). This result was supported by the highest contamination of leafy greens with *Blastocystis* (35.9%) ( *P*=0.0064), *Eimeria* (33.5%) ( *P*=0.0063), *Balantidium* (15.1%) ( *P*<0.0001), *Entamoeba* spp. (16.8%) ( *P*=0.0021) and *Giardia* (12.6%) ( *P*=0.0290). However, vegetables were found to be more contaminated by helminths than leafy greens (24.2%) ( *P*=0.0082), determined by Strongylida (23.6%) ( *P*=0.0150).

**Table 6.** Comparation of parasitic contamination between vegetables and leafy greens.

Parasites	Vegetables			Leafy Greens		
	Total			Total		
	n=488	%	IC	n=412	%	IC
*Blastocystis* sp.	134	27.5	(23.5-31.4)	148	35.9	(31.3-40.6)
*Entamoeba* spp.	48	9.8	(7.2-12.5)	69	16.8	(13.1-20.4)
*E. coli*	2	0.4	(0-1)	1	0.2	(0-0.7)
*E. hartmanni*	11	2.3	(0.9-3.6)	3	0.7	(0-1.6)
*Endolimax nana*	2	0.4	(0-1)	1	0.2	(0-0.7)
*Iodamoeba buetschlii*	9	1.8	(0.7-3.0	15	3.6	(1.8-5.5)
*Giardia* spp.	40	8.2	(5.8-10.6)	52	12.6	(9.4-15.8)
*Chilomastix* spp.	8	1.6	(0.5-2.8)	3	0.7	(0-1.6)
*Retortamonas* spp.	1	0.2	(0-0.6)	0	0	(0-0)
*Enteromonas* spp.	0	0	(0-0)	1	0.2	(0-0.7)
*Cryptosporidium* spp.	30	6.2	(4.0-8.3)	24	5.8	(3.6-8.1)
*Cyclospora* spp.	20	4.1	(2.3-5.9)	12	2.9	(1.3-4.5)
*Cystoisospora* spp.	2	0.4	(0-1)	2	0.5	(0-1.2)
*Eimeria* spp.	123	25.2	(21.4-29.1)	138	33.5	(28.9-38.1)
*Balantidium* spp.	30	6.2	(4.0-8.3)	62	15.1	(11.6-18.5)
Protozoa	230	47.1	(42.7-51.6)	253	61.4	(56.7-66.1)
*Ascaris* spp.	3	0.6	(0-1.3)	2	0.5	(0-1.2)
Strongylida	115	23.6	(20.4-28)	70	17	(13.4-20.6)
Helminths	118	24.2	(20.4-28)	70	17	(13.4-20.6)
Total	331	67.8	(63.7-72)	317	76.9	(72.8-81.0)

## Discussion

The results of the present study prove that the fruits, vegetables and green leafy that are cultivated and harvested in the capital of San Andrés, the area with the highest agricultural production in the Ecuadorian Andes, present significant contamination with parasites. Multiparasitism in the samples analyzed reflects inadequate hygiene conditions during agricultural activities and the crops products thus obtained represent a possible vehicle for parasites when consumed without adequate sanitation. It is important to note that agricultural production is marketed locally, regionally, nationally, and internationally; therefore, the risk of contagion to individuals is extrapolated to non-endemic areas.

Direct contamination with human and animal excrements is a potential source of contamination of anthroponotic and zoonotic parasites for vegetables, so their consumption constitutes an important risk factor associated with the transmission of infective forms. However, it is possible that free-living parasites (Strongylida), also contaminate these crop products, being considered an insignificant finding, in this population where our research group has detected parasite prevalence’s reaching 97.3% in humans ( González-Ramírez *et al*., 2022) and 90.3% in animals ( González-Ramírez *et al*., 2021).

When contrasting the results of the present investigation carried out in the capital of San Andrés with those obtained in six different communities of the same parish, the total percentage of contamination is lower in agricultural products harvested in the capital (63.4%) compared to the other communities further away, located at higher altitudes and with a larger indigenous population (70.6%); likewise, fruits (48.4 *versus* 67.1%) and vegetables (67.8 *versus* 73.6%) ( González-Ramírez *et al*., 2022). These differences may be present because the capital of San Andrés offers better environmental sanitation conditions and the farmers have a higher level of education and better access to the urban area.

In the present study, leafy greens were more contaminated (76.9%) than vegetables (67.8%) and fruits (48.4%), likely because these maintain contact with the soil and organic fertilizers from the beginning as seedlings until they are fully grown, and external leaves allow protection for internal plant parts in contact with contaminated soil. The greater parasitic contamination of leafy greens has been explained by the irregularities of their leaves and the roughness of their surface that allows the adhesion of infectious parasitic forms that persist in the environment ( Vuong *et al*., 2007; Allende *et al*., 2017).

Vegetables were the second most contaminated products after leafy greens, which is explained by the greater contact they maintain with the soil. The rooted vegetables (tubercle) were found to be more parasitized by nematodes (24.3%), always being less than contamination by protozoa (47.1%), possibly because they grew under the ground. It is important to highlight that, onions, carrots and radishes are frequently consumed raw and can function as efficient vehicles for parasites.

In creeping fruits such as strawberries, a greater number of contaminating parasitic species was found, compared to those that grow on shrubs and trees, perhaps due to direct contact with irrigation water ( Esteban *et al*., 2002; Daniels *et al*., 2016; González-Ramírez *et al*., 2020), organic fertilizer and the soil ( Dixon, 2016; Rodríguez-Eugenio *et al*., 2019).

In addition, the roughness of its surface is a condition that can also influence in the contamination of blackberry and peaches ( Resendiz-Nava *et al*., 2020). Although, these rough fruits do not come into contact with the soil, nor with irrigation water, the texture of their surface allows the adhesion of parasites dispersed by the wind, insects or the hands of farmers, as explained by Dixon (2016) and Machado-Moreira *et al*. (2019).

Animal faeces is a source of nutrients for the soil, fertilizing agro-systems at low cost ( Daniels *et al*., 2016), but if this material does not receive prior treatment, it is highly polluting. Among the inappropriate agricultural practices detected in San Andrés, considered risk factors, the fertilization of crops with fresh excreta from parasitized animals, as well as the contamination derived from their displacement on the crops, dispersing viable parasitic infectious forms persist in the environment ( Gutiérrez-Rodríguez and Adhikari, 2018; Julien-Javaux *et al*., 2019; Resendiz-Nava *et al*., 2020; González-Ramírez *et al*., 2021). Additionally, the contamination of soils with human fecal matter contained in septic tanks that overflow or leak is another important source of contamination ( Daniels *et al*., 2016; González-Ramírez *et al*., 2022).

On the other hand, the irrigation of crops with water bodies conducted by channels or contained in artificial wells that receive runoff from rain should be considered a risk factor for parasitic dispersal, as has been verified by Machado-Moreira *et al*. (2019) and González-Ramírez *et al*. (2020). These artificial water resources carry a high risk to human health in relation to the spread and increased transmission of parasites as shown by Esteban *et al*., (2002). Our results question the rationality of irrigation projects through open channels that carry contaminated water and artificial wells from which the animals drink. Furthermore, this water is used for the dilution of fertilizers and fungicides, machinery and the washing of work equipment and utensils that increase the possibility of contamination of vegetables ( Dixon, 2016; Machado-Moreira *et al*., 2019; Trelis *et al*., 2022).

In addition, aspects of the field that promote the dissemination of parasitic forms found in the soil include flooding, rain, and sprinkler irrigation that allow the transport of microorganisms from the soil to the plants, as confirmed by Efstratiou *et al*. (2017), or when water drops splash as explained by Dixon (2016).

Likewise, the wind brings dust particles from the ground that aid adherence of parasitic forms to the vegetables or fruits of trees or shrubs ( Dixon, 2016; Machado-Moreira *et al*., 2019), which explains the finding of Strongylida on the woolly surface of peaches. Additionally, insects, rodents, wild animals and the contaminated hands of farmers can spread parasites, as indicated by Dixon (2016), Machado-Moreira *et al*. (2019) and González-Ramírez *et al*. (2021).

Moreover, various actions carried out by farmers can contaminate crop products, including handling of vegetables without hygienic measures by the personnel in charge of sowing and harvesting, as has been verified in various localities ( Dixon, 2016; Machado-Moreira *et al*., 2019; Li *et al*., 2020). However, the transfer, storage, washing, packaging, distribution, and marketing activities that are carried out after harvest also contribute to food contamination ( Etewa *et al*., 2017; Trelis *et al*., 2022).

After becoming aware of the parasitic contamination of food grown in the area, consumers from any part of the world should be warned that they must properly sanitize fruits, vegetables and leafy greens before consuming them, especially those that are eaten raw, if its origin is unknown. Just as this alarming contamination has been detected in this agricultural area, it is likely that it also occurs in other rural areas, mainly in low-income countries where producers do not apply hygienic measures during their agricultural practices, as previously was demonstrated by Pérez-Cordón (2008) in the Andean zone of Peru.

The potential effects of primary production activities on food safety need to be considered. These include identifying any specific points where the probability of contamination may exist and taking specific measures to minimize them. Growers are required to implement measures to prevent contamination of air, soil, water, feed, fertilizers, pesticides, or any other agent used in production and to control animal health so that it does not pose threats. If programs are implemented and executed to guarantee sanitary control in the farms and the objectives of food security are achieved in primary production, exports would increase, translating to an increase in the economic income of the producing countries.

The considerable parasitic contamination in vegetables obtained in the field immediately after harvest in this zone might be one of the causes of the high parasitic prevalence in humans (98.2%), mechanical vectors (52.7%) ( González-Ramírez *et al*., 2022), and animals (90.3%) ( González-Ramírez *et al*., 2021), as well as the 100% contamination of man-made water resources (channels and wells) of these rural communities ( González-Ramírez *et al*., 2020).

These results suggest the need to integrate protozoa and helminths into the list of contaminants that are handled in the microbiological criteria required by the Ecuadorian Technical Standard ( INEN, 2016). Monitoring only *Escherichia coli* in vegetables and fruits is not a good indicator of the absence of faecal contamination, nor does it guarantee food safety. Protozoa, whose high resistance to temperatures and disinfectants ( Ramos *et al*., 2013) and low infectious doses have been demonstrated constitute a significant risk for consumers.

Policy decisions should promote the development of mitigation plans that involve health and hygiene education programs for producers and consumers. In addition, more advanced technological procedures and treatments that contribute to contamination prevention, as well as the inactivation and elimination of infectious forms in contaminated fresh produce to improve the quality and safety of these foods in accordance with the standards of Caradonna *et al*. (2017), should be promoted.

The sedimentation technique, Ziehl Neelsen staining, and measurement with the ocular micrometer performed for parasitic detection in the present study allowed us to carry out a low-cost analysis, as long as, microscopic visualization is done by trained analysts, fresh plant products can be monitored in other endemics areas of developing countries, where biological analysis cannot be performed by molecular techniques because of its high cost. We are aware of the importance of determining the parasitic species by molecular methods for epidemiological control. However, for surveillance studies on the contamination of these products in poor countries, microscopic diagnosis (though insufficient) is relevant because it is the only thing available; these results provide the basis for food safety guidelines to reduce the risk of contamination and minimize the transmission of food-borne parasitic diseases.

The samples of vegetables and fruits were analyzed by light microscopy alone because of limited resources to perform molecular analysis and the difficulty in obtaining permission to transport the samples to a molecular laboratory, but the overall prevalence detected in this study was one of the highest described thus far. In this Andean region of Ecuador, the global contamination of agricultural products by parasites has a mean prevalence of 63.4 and 70.6% ( González-Ramírez *et al*., 2022), which are higher than those described in Phnom Penh, Cambodia 56.0% ( Vuong *et al*., 2007), Alexandria, Egypt 31.7% ( El Said Said, 2012), Koforidua, Ghana 57.5% ( Kudah *et al*., 2018), Arba Minch, Ethiopia 54.4% ( Bekele *et al*., 2017), Nakhon Si Thammarat, Thailand 35.1% ( Punsawad *et al*., 2019), and Damascus, Syria 34.4% ( Al Nahhas and Aboualchamat, 2020).

Nevertheless, our results are similar to those found in Trujillo, Peru, where Pérez-Cordón *et al*. (2008) reported the presence of *Giardia*, *Cyclospora*, *E. nana*, *Iodamoeba buetschlii*, *Blastocystis* and *Ascaris lumbricoides.* Moreover, the prevalence values are similar to those reported in Mina Gerais, Brazil, by Luz *et al*. (2017), with 50.9% of vegetables contaminated, with a predominance of nematode larvae (36.5%), *Entamoeba coli* (26.0%) and eggs of hookworms/ *Strongyloides* spp. (12.9%).

Our results also differ from those obtained by Honório Santos *et al*. (2019) in Bahia, Brazil, with prevalence’s of 70% in fruits: guava (90%), lemon and apple (70%) and grape (50%). The highest prevalence in this study was of the helminths *A. lumbricoides,* Ancylostomids, *Taenia* spp., and *Enterobius vermicularis*, followed by the protozoa *Balantidium coli* and *Entamoeba coli.* These differences might be due to the high altitude of San Andrés, where the evolution of soil-transmitted helminths is limited.

Interestingly, in San Andrés, there were significant differences between contamination in leafy green types, which is consistent with the results of other studies that indicate that the highest-contaminated vegetable is lettuce, reaching rates of 29.5% in Damascus ( Al Nahhas and Aboualchamat, 2020), 54.2% in Ghana ( Kudah *et al*., 2018), and 61.1% in Mina Gerais, Brazil ( Luz *et al*., 2017).

Food-borne transmission of protozoan parasites is an emerging issue in developed countries around the world. *Giardia*, *Cryptosporidium* and *Cyclospora* have been implicated in both human and animal illness: unpasteurized apple juice, unwashed onions, salad, mixed baby lettuce, basil, sandwiches, fruit salad and raspberries ( Dixon, 2016). Rzezutka *et al*. (2010), in Lublin, Poland, detected *Cryptosporidium* sp. in 4.7% of fresh vegetables; in packaged salads, Italy revealed 4.2% contamination of the samples, and the prevalence of each species was for *G. duodenalis* 0.6% *, T. gondii* 0.8% *, Cryptosporidium* spp. 0.9%, *C. cayetanensis* 1.3%, *B. hominis* 0.5% and *D. fragilis* 0.2% ( Caradonna *et al*., 2017). In contrast, Trelis *et al*. (2022) were able to prove higher contamination with *G. duodenalis* 23.3% and *Cryptosporidium* spp. 7.8% in green leafy vegetables marketed in the city of Valencia, Spain.

Information collected at each sampling point checked to field cultivation as the critical step for contamination ( Luz *et al*., 2017). The high parasitic frequency is associated with the inadequate handling of crop products, as well as, to the inefficient sanitary conditions of the places where they are marketed. It is recommended to teach hygienic measures through sanitary education for farmers, merchants, and consumers ( Honório Santos *et al*., 2019).

In these tropical countries, the highest records of parasitic contamination are in vegetables, so they are described as endemic for enteric parasites. From there, they are spread to other countries through fresh vegetables. Developing countries have not been able to control their enteric-parasitosis because of the low socioeconomic and hygienic-sanitary levels, inability to offer adequate sanitary infrastructures and the education that could change of habits in people and prevent soil, water, and food contamination.

The implementation of control measures in fresh produce preharvest and postharvest, as well as an adequate sanitary hygienic level of the producer, handler, and consumer, will be crucial to minimize the food transmission of protozoa and helminths. To control parasites at the time of cultivation and harvest, irrigation with properly treated water, monitoring the health and hygiene of agricultural workers, improving agricultural sanitation, and restricting access of livestock and other animals to crops and surface water bodies (building adequate drinking troughs) are needed. Additionally, proper construction and maintenance of septic tanks is important to prevent contamination by overflow.

Unsafe agricultural practices, such as irrigation with untreated contaminated water and fertilization of the soil with improperly treated animal manure, are used very commonly by small farmers; mainly in developing countries, due to the export of agricultural products. To mitigate this problem, it is necessary to use treated water for irrigation, washing fresh produce, washing hands and equipment. Good hygienic practices by farm workers involved in the cultivation, harvesting and handling of fresh produce are another important means of reducing the likelihood of contamination at the farm level in endemic regions.

## Conclusion

This research demonstrated the important parasitic contamination of fruits, vegetables, and leafy greens. Warns about the risk of consuming raw products from these crops, without proper hygiene can be infection source of enteroparasites to humans and animals in this area or in nonendemic areas where these products are marketed. This study establishes the need for strict hygienic measures in growing and harvest areas, which can be achieved by the treatment of soil, manure, and water used for the cultivation of vegetables and fruits, as well as proper disinfection before consumption.

## Data Availability

Figshare: Parasitic contamination of fruits, vegetables and leafy greens harvested in an Andean agricultural area, https://doi.org/10.6084/m9.figshare.22313335.v2 ( González-Ramírez *et al*., 2023). This project contains the following underlying data:
• Data parasites fruits vegetables Ecuador.xlsx Data are available under the terms of the Creative Commons Zero “No rights reserved” data waiver (CC0 1.0 Public domain dedication).
