# Field study of parasitic contamination of fruits, vegetables and leafy greens in the Ecuadorian Andes

**DOI:** 10.12688/f1000research.132957.2

**Published:** 2024-03-12

**Authors:** Luisa Carolina González-Ramírez, Pablo Djabayan-Djibeyan, José G. Prato, Cecilia Alejandra García Ríos, Julio César Carrero, María Trelis, Màrius Vicent Fuentes

**Affiliations:** 1 Grupo de Investigación "Análisis de Muestras Biológicas y Forenses", Carrera de Laboratorio Clínico, Facultad de Ciencias de la Salud, Universidad Nacional de Chimborazo, Riobamba, Chimborazo Province, 060103, Ecuador; 2 Grupo de Investigación "Salud Pública", Carrera de Medicina, Facultad de Ciencias de la Salud, Universidad Nacional de Chimborazo, Riobamba, Chimborazo Province, 060103, Ecuador; 3 Grupo de Investigación “Estudios Interdisciplinarios”, Ingeniería Ambiental, Facultad de Ingeniería, Universidad Nacional de Chimborazo, Riobamba, Chimborazo Province, 060103, Ecuador; 4 Facultad de Salud Pública, Escuela Superior Politecnica de Chimborazo, Riobamba, Chimborazo Province, 060103, Ecuador; 5 Departamento de Inmunología, Instituto de Investigaciones Biomédicas, Universidad Nacional Autónoma de México, Mexico City, Mexico City, 04510, Mexico; 6 Research Group "Parasites and Health", Departament de Farmàcia i Tecnologia Farmacèutica i Parasitologia, Facultat de Farmàcia, Universitat de València, Burjassot/Valencia, Comunidad Valenciana, 46010, Spain; 7 Joint Research Unit on Endocrinology, Nutrition and Clinical Dietetics, Universitat de València - Health Research Institute La Fe (IISLAFE), Valencia, Valencian Community, 46026, Spain

**Keywords:** agricultural production, food, transmission, parasites, fruits, vegetables, leafy greens

## Abstract

**Background:**

Raw vegetables have been considered vehicles of enteroparasites. South American countries are among the most important exporters of fresh vegetables, including Ecuador, which has a tropical climate and soils rich in organic matter that allow it to harvest throughout the year for sale to different countries. The aim of the study was to assess the occurrence of the parasitic contamination of fruits, vegetables and leafy greens grown in an agricultural area of the Ecuadorian Andes.

**Methods:**

A cross-sectional field study was conducted with snowball sampling on 1,416 samples (516 fruits, 488 vegetables, and 412 leafy greens). Each sample were washed with water, and the resulting solution after removing the vegetables, was subjected to 24-hour sedimentation. The concentrated sediment underwent microscopic analysis.

**Results:**

The overall positivity for parasitic contamination was 63.4%, with leafy greens having the highest contamination rate (76.9%) (P<0.0001), surpassing vegetables (67.8%) and fruits (48.4%). Cabbage (100%), onions (84%), and strawberries (60.2%) emerged as the most contaminated within their respective groups. Protozoa were more prevalent (49.6%) than helminths (15.5%) (P<0.0001). *Blastocystis* sp. (33.5%) ranked highest, followed by *Eimeria* spp. (26.3%), *Entamoeba* spp. (10.3%), *Giardia* spp. (8.3%), *Balantidium* spp. (6.9%), *Cryptosporidium* spp. (6.6%), *Cyclospora* spp. (4.4%), *Cystoisospora* spp. (0.5%), Strongylida (15.5%), and *Ascaris* spp. (0.4%).

**Conclusions:**

The study reveals that vegetables and fruits for human consumption from this area of the Ecuadorian Andes are highly contaminated with various parasites, constituting a possible source of infection for humans and animals in this area, or in non-endemic areas where these products are marketed. The finding emphasizes the need for strict hygienic measures in agricultural crops, which will be properly achieved through the treatment of soil, manure and water used for cultivation.

## Introduction

Incorporating vegetables, leafy greens, and fruits into human diet is essential to ensure vital nutrients crucial to maintaining health. However, these foods can also serve as vehicles for enteroparasites, representing a paradox within nutritional health practices ( Punsawad et al., 2019; Al Nahhas and Aboualchamat, 2020; Barlaam et al., 2021, 2022; Faria et al., 2023). The role of vegetables in the spread of pathogens is notably substantial. The external surfaces of these foods can retain infectious stages of various parasites, thereby posing a risk of direct transmission to humans when consumed raw or poorly washed ( Mufida et al., 2022; Lucas et al., 2023; Moreno-Mesonero et al., 2023).

The importation of fresh vegetables from endemic to non-endemic regions has contributed to the spread of parasites. Diarrhoea epidemics have been reported from the consumption of berries, tomatoes, peppers, onions, carrots, lettuces, cabbage, radishes and mixed-salad packages ( Dixon, 2016; Machado-Moreira et al., 2019; Barlaam et al., 2021, 2022; Temesgen et al. 2022). Leafy greens are highlighted by the potential role of spinach, lettuce, cabbage, watercress, basil, mint, coriander, and parsley as vehicles for food-borne parasites ( Ahlinder et al., 2022; Lucas et al., 2023; Moreno-Mesonero et al., 2023). There is evidence that leafy green can carry and spread parasites as *Cyclospora cayetanensis*, *Cryptosporidium* spp. and *Giardia duodenalis*, *Toxoplasma gondii*, *Entamoeba histolytica*, *Blastocystis* sp., *Cystoisospora belli*, *Balantidium coli*, *Dientamoeba fragilis*, *Echinococcus* sp., *Dipylidium caninum*, *Ascaris* sp., *Trichuris* sp., and Nematode spp. Larvae ( Dixon, 2016; Caradonna et al., 2017; Karshima, 2018; Robertson, 2018; Barlaam et al., 2021; Yahia et al., 2023).

The persistence and survival of parasites in soil ( Qorom, et al. 2023; Falcone et al., 2023), vegetables ( Falcone et al., 2023) and water ( Kubina et al. 2023), has been proven. According to these studies, *Cryptosporidium* survives in lamb’s lettuce for two months and its washing showed limited effectiveness in reducing parasite load and had no impact on the parasite’s survival. Furthermore, chlorination of the wash water failed to improve the efficiency of the disinfection process. The situation is aggravated by cases of parasite resistance to certain chemical and physical inactivating agents ( Ramos et al., 2013). This underlines the ability of parasites to persist and survive along the food chain, facilitating their transmission to humans, even far from the site of production ( Dixon, 2016; Caradonna et al., 2017; Barlaam et al., 2021, 2022; Temesgen et al. 2022; Ahart et al., 2023).

Fruits are also considered significant carriers of parasites because they are consumed raw and do not undergo disinfection treatments such as the use of vinegar ( Honório Santos et al., 2019). The study of berries is particularly important since their consumption has increased recently due to their high nutritional value, as a source of bioactive compounds and antioxidants ( Tefera et al., 2018; Barlaam et al., 2022; Lucas et al., 2023). Unfortunately, berries such as raspberries and strawberries can carry infective forms of pathogens due to their delicate and porous nature, facilitating the attachment and protection of parasites. Inappropriate practices in cultivation, harvesting and handling pose a significant risk to consumers ( Tefera et al., 2018; Temesgen et al., 2022). Recent molecular studies demonstrated the persistence in berries of parasites such as *Cryptosporidium*, *Cyclospora*, *Giardia duodenalis*, *Entamoeba histolytica*, *Toxoplasma gondii*, *Acanthamoeba*, *Vermamoeba vermiformis*, *Blastocystis* sp., and *Echinococcus* ( Marques et al., 2020; Barlaam et al., 2021, 2022; Trelis et al., 2022; Temesgen et al., 2022; Moreno-Mesonero et al., 2023), highlighting their resistance to washing processes and disinfection ( Temesgen et al., 2022; Kubina et al., 2023).

The presence of parasites in the vegetables is an indicator of the lack of adherence to good agricultural practices ( Lucas et al., 2023). The main risk factors for the transmission of parasites through vegetables include the soil contamination with excrement from defecation or direct fertilization ( Ercumen et al., 2017; Falcone et al., 2023) and the use of contaminated water for irrigation, pesticide dilution or equipment washing ( Efstratiou et al., 2017; Karshima, 2018; Tefera et al., 2018).

Although parasites do not multiply in food, the transmission of infectious forms is closely linked to their resistance to survive in the environment attached to vegetables, as reported by studies carried out throughout Latin America: Argentina ( Falcone et al., 2023); Brazil ( Luz et al. 2017; Honório Santos et al., 2019; De Farias et al., 2021); Bolivia ( Rodríguez et al., 2015); Colombia ( Polo et al., 2016); Cuba ( Puig-Peña et al., 2013); Ecuador ( Bracho-Mora et al., 2022); Peru ( Pérez-Cordón et al., 2008; Benites Salcedo et al., 2019; Lucas et al., 2023); Mexico ( Chávez-Ruvalcaba et al., 2021); Venezuela ( Cazorla-Perfetti et al., 2013; Devera et al., 2021).

South American countries are among the most important exporters of fresh vegetables. Ecuador has tropical climate and soils rich in organic matter that allow it to harvest fruits, vegetables, and grains throughout the year. According to data from the Agriculture and Livestock Ministry, during 2014-2018 period, Ecuador raised more than $3,500 million by exporting 6 million tons of fruits and vegetables (Ministerio de Agricultura y Ganadería Ecuador, 2020). Unfortunately, Ecuador has serious health problems in rural Andean regions, especially those located at high altitudes, mostly inhabited by indigenous populations whose means of subsistence is agriculture, livestock and animal husbandry ( González-Ramírez et al., 2021, 2022).

Moreover, Ecuadorian farmers often do not use good agricultural practices due to the lack of training, confidence, or economic resources, which is detrimental to food quality production. Two local reports have shown high level of parasitic contamination in vegetables: up to 82.3% in lettuce from Manabí province ( Bracho-Mora et al., 2022) and up to 70.6% in fruits and vegetables of six rural communities in the parish of San Andrés, Chimborazo province ( González-Ramírez et al., 2022).

Due to the alarming contamination data previously reported by our group, in this work we have evaluated the detailed parasitic contamination of all fruits, vegetables and leafy greens grown in the capital of San Andrés, an agricultural zone of the Ecuadorian Andes.

## Methods

### Study area

The study area was the community of San Andrés, Guano canton, Chimborazo province of Ecuador, located at 3,900 meters above sea level. The local temperature ranges between 5-18 °C, and rainfall varies between 500-1,000 mm/year. There are two rainy periods, February to May and October to November; the remaining months are transitional with moderate rains. Evapotranspiration affects the drought of the soil, which originates from volcanic ashes of variable textures, most of which are shallow silty loam, with a pH of 4.5 to 6.5. There are loamy soils in the areas with the highest agricultural production, but they are affected by chemical fertilizers. There are also sandy soils with low fertility because they do not retain moisture and nutrients; the latter and the action of steep slopes make them susceptible to erosive processes; consequently, crops and sowing grass are not abundant. However, agricultural activity is 34.5%, and cattle breeding activity is 50.4%; these two are the main means of financial income for the local population ( PDOT San Andrés, 2015).

Government records indicate that 47.9% of the rural population of Ecuador lives in poverty, with an average monthly family income of $84.05, and 27.5% living in extreme poverty, with an average income of $47.70. The province of Chimborazo has an illiteracy rate of 13.5%, and the community of San Andrés has an indigenous population of 36.9% ( INEC, 2020). Hence, their training is based on habits and customs acquired from their ancestors, which may contribute to as a lack of basic hygiene and sanitary measures. The most remote communities have built septic tanks, and the communities closest to the capital have sewers; however, both drain wastewater into rivers and streams ( PDOT San Andrés, 2015).

### Investigation design

A field study, cross-sectional, observational and descriptive, was carried out during 1 month of rain and 7 months of drought. The snowball sampling technique was applied, whereby a grower helped locate the nearest farm and so on. All types of products found were included in the sampling (1,416 samples in total); the inclusion criteria were that all agricultural products must come from San Andrés fields and those not cultivated in the community were excluded.

### Sampling

The total of 1,416 samples analyzed included 516 fruits of 8 types: *Fragaria ananassa* (strawberry), *Rubus glaucus* (blackberry), *Physalis peruviana* (uvilla), *Prunus persica* (peach), *Citrus limon* (lemon), *Psidium guajava* (guava), *Ficus carica* (fig), and *Solanum lycopersicum* (tomato); 488 vegetables of 9 types: *Allium cepa* var. rosum (red onions) and *Allium cepa L* (white onions), *Solanum tuberosum* (potato), *Daucus carota* (carrot), *Raphanus sativus* (radish), *Beta vulgaris* (beet), *Capsicum annuum* (sweet pepper), *Capsicum frutescens* (chili pepper), and *Lupinus mutabilis* (bean chochos) and 412 leafy greens of 8 types: *Medicago sativa* (alfalfa), *Lactuca sativa* (lettuce), *Brassica oleracea* (cabbage), *Beta vulgaris* (chard), *Petroselinum crispum* (parsley), *Coriandrum sativum* (cilantro), *Apium graveolens* (celery), and *Nasturtium officinale* (watercress).

All samples were obtained from the owners’ fields and stored in hermetically sealed propylene bags. Each sample was labelled indicating the plant species name, origin, date, and time of collection. The samples were immediately transported in their containers with cooling gels to the Laboratorio de Investigación de la Facultad de Ciencias de la Salud, Universidad Nacional de Chimborazo, to be processed within one hour of collection.

### Ethical considerations

The sampling was carried out with the appropriate permission of the Cantonal and Parochial Decentralized Autonomous Governments. All farmers collected samples of their own crops (as they always do), knowing that the study benefits the community, without compromising the health of the population with respect to bioethical principles.

### Parasitological analysis

The processing protocol for the parasitological analysis of all samples, previously described by Rivero de Rodríguez *et al*. (1998), was utilized. For the processing of the samples, 75 g of vegetables, fruits or green leaves were taken and added to 500 mL of previously filtered and boiled water. The contents were stirred with the help of a magnetic stirrer for 1 hour, the remains of the vegetable were removed and the solution was left to stand for 24 hours. Subsequently, the solution was decanted and the first fraction was collected in 15 mL tubes to be subjected to centrifugation for 5 min at 800 xg. Once the concentrate or sediment was separated, the supernatant was discarded and the precipitate was reconstituted in 400 μL of saline (0.85%). Each sample was observed under a light microscope (Nikon E200) using 10x and 40x objectives. In addition, iodized solution and the ocular micrometer were used when necessary, for stain parasitic structures or to measure the dimensions for their recognition. Additionally, a smear was made with one drop from the pellet and prepared for acid-fast staining (using a modified Zielh-Neelsen technique) for coccidia oocyst detection and identification after measurement, mainly *Crytosporidium* and *Cyclospora*, and subsequent microscopic assessment (100×) ( García *et al*., 1983).

### Statistical analysis

The database made in Microsoft Excel was exported to SPSS Statistic 26.0 software (IBM, New York, NY, USA). The difference in parasitic contamination between the various categories of plant products and the predominant parasite type in each plant species were compared using Pearson’s chi-square test (χ ^2^) and Fisher’s exact test, when appropriate. A *P* value <0.05 was considered statistically significant.

## Results

When analyzing the different crop products, a total of 898 (63.4%) were contaminated by parasites. Noteworthy, every sample analyzed showed more than one associated parasite, i.e. 100% multiparasitism was detected. A statistically significant difference between the overall contamination rates, was observed with the leafy greens (76.9%) being more contaminated than vegetables (67.8%) and both, more contaminated than fruits (48.4%) ( *P*<0.0001). In total, 15 protozoa and 2 helminth nematodes were identified, with protozoa also showing a higher prevalence (49.6%) than nematodes (15.5%) ( *P*<0.0001). *Blastocystis* sp. was outstanding (33.5%) ( *P*<0.0001), showing central body, granular, and resistance forms, whereas dividing, globular, or amoeboid forms were not observed. Other protozoa identified include *Eimeria* spp. (26.3%), *Entamoeba* spp. (10.3%), *Giardia* spp. (8.3%) and *Cryptosporidium* spp. (6.6%). Regarding the nematodes, Strongylida was more frequent than *Ascaris* spp. ( *P*<0.0001) (see Table 1).

**Table 1.** Distribution of parasites according to the plant type analysed.

Parasites	Fruits			Vegetables			Leafy Greens			Total		
	n=516	%	IC	n=488	%	IC	n=412	%	IC	n=1416	%	IC
*Blastocystis* sp.	193	37.4	(33.2-41.6)	134	27.5	(23.5-31.4)	148	35.9	(31.3-40.6)	475	33.5	(31.1-36)
*Entamoeba* spp.	29	5.6	(3.6-7.6)	48	9.8	(7.2-12.5)	69	16.7	(13.1-20.4)	146	10.3	(8.7-11.9)
*E. coli*	9	1.7	(0.6-2.9)	11	2.3	(0.9-3.6)	3	0.7	(0-1.6)	23	1.6	(1-2.3)
*E. hartmanni*	2	0.4	(0-0.9)	2	0.4	(0-1)	1	0.2	(0-0.7)	5	0.4	(0-0.7)
*Endolimax nana*	31	6	(4.0-8.1)	9	1.8	(0.7-3.0)	15	3.6	(1.8-5.5)	55	3.9	(2.9-4.9)
*Iodamoeba buetschlii*	2	0.4	(0-0.9)	2	0.4	(0-1)	1	0.2	(0-0.7)	5	0.4	(0-0.7)
*Giardia* spp.	26	5	(3.2-7)	40	8.2	(5.8-10.6)	52	12.6	(9.4-15.8)	118	8.3	(6.9-9.8)
*Chilomastix* spp.	6	1.2	(0.3-2.1)	8	1.6	(0.5-2.8)	3	0.7	(0-1.6)	17	1.2	(0.6-1.8)
*Retortamonas* spp.	0	0	0	1	0.2	(0-0.6)	0	0	0	1	0.1	(0-0.2)
*Enteromonas* spp.	3	0.6	(0-1.3)	0	0	0	1	0.2	(0-0.7)	4	0.3	(0-0.6)
*Cryptosporidium* spp.	39	7.6	(5.3-9.9)	30	6.1	(4.0-8.3)	24	5.8	(3.6-8.1)	93	6.6	(5.3-7.9)
*Cyclospora* spp.	31	6	(4.0-8.1)	20	4.1	(2.3-5.9)	12	2.9	(1.3-4.5)	63	4.4	(3.4-5.5)
*Cystoisospora* spp.	3	0.6	(0-1.2)	2	0.4	(0-1)	2	0.5	(0-1.2)	7	0.5	(0.1-0.9)
*Eimeria* spp.	112	21.7	(18.1-25.3)	123	25.2	(21.4-29.1)	138	33.5	(28.9-38.1)	373	26.3	(24.0-28.6)
*Balantidium* spp.	5	1	(0.1-1.8)	30	6.1	(4.0-8.3)	62	15	(11.6-18.5)	97	6.9	(5.5-8.2)
Protozoa	220	42.6	(38.4-46.9)	230	47.1	(42.7-51.6)	253	61.4	(56.7-66.1)	703	49.6	(47-52.3)
*Ascaris* spp.	0	0	0	3	0.6	(0-1.3)	2	0.5	(0-1.2)	5	0.4	(0-0.7)
Strongylida	32	6.2	(4.1-8.3)	115	23.6	(20.4-28.0)	70	17	(13.4-20.6)	217	15.3	(13.7-17.4)
Helminths	32	6.2	(4.1-8.3)	118	24.2	(20.4-28.0)	70	17	(13.4-20.6)	220	15.5	(13.7-17.4)
Total	250	48.4	(44.1-52.7)	331	67.8	(63.7-71.9)	317	76.9	(72.8-81)	898	63.4	(60.9-65.9)

n = number of studied; IC = Confidence interval.

When analyzing the percentages of parasites in the three groups of samples, the statistical analysis revealed a high prevalence in fruits of *Blastocystis* (37.4%) ( *P*=0.0018), *Cryptosporidium* (7.6%) ( *P*<0.0001), *Cyclospora* (6%) ( *P*<0.0001) and *Endolimax nana* (6%) ( *P*=0.0028). In contrast, vegetables were mostly contaminated by helminths (24.2%) ( *P*<0.0001), represented mainly Strongylida (23.6%) ( *P*<0.0001). Finally, the leafy greens showed high contamination with *Eimeria* (33.5%) ( *P*=0.0002), *Entamoeba* spp. (16.7%) ( *P*<0.0001), *Balantidium* (15.0%) ( *P*<0.0001) and *Giardia* (12.6%) ( *P*=0.0002). Overall, a total parasitic contamination of 76.9% ( *P*<0.0001) with 61.4% ( *P*<0.0001) being protozoa was obtained (see Table 1).

Table 2 summarizes the results according to the type of fruit, the highest number of protozoa was found in strawberries (60.2%) ( *P*<0.0001), with *Blastocystis* sp. (59.2%) ( *P*<0.0001), *E. nana* (17.4%) ( *P*<0.0001) and *Cyclospora* spp. (14.3%) ( *P*=0.0011) the most frequent. In contrast, peaches were more often contaminated with helminths (30%) ( *P*<0.0001).

**Table 2.** Distribution of parasites according to the fruit type analysed.

Parasites	Strawberry		Blackberry		Uvilla		Peach		Lemon		Guava		Fig		Tomato		Total	
	n=98	%	n=83	%	n=56	%	n=50	%	n=56	%	n=57	%	n=50	%	n=66	%	n=516	%
*Blastocystis* sp.	58	59.2	35	42.2	19	33.9	24	48	14	25	19	33.3	15	30	9	13.6	193	37.4
*Entamoeba* spp.	4	4.1	5	6	0	0	2	4	0	0	9	15.8	3	6	6	9.1	29	5.6
*E. coli*	4	4.1	4	4.8	0	0	0	0	0	0	1	1.8	0	0	0	0	9	1.7
*E. hartmanni*	0	0	1	1.2	0	0	0	0	0	0	0	0	0	0	1	1.5	2	0.4
*Endolimax nana*	17	17.3	6	7.2	0	0	3	6	1	1.8	3	5.3	0	0	1	1.5	31	6
*Iodamoeba buetschlii*	0	0	0	0	0	0	0	0	0	0	0	0	0	0	2	3	2	0.4
*Giardia* spp.	10	10.2	4	4.8	1	1.8	1	2	1	1.8	1	1.8	2	4	6	9.1	26	5
*Chilomastix* spp.	0	0	3	3.6	0	0	0	0	0	0	0	0	2	4	1	1.5	6	1.2
*Retortamonas* spp.	3	3.1	0	0	0	0	0	0	0	0	0	0	0	0	0	0	3	0.6
*Enteromonas* spp.	0	0	0	0	0	0	0	0	0	0	0	0	0	0	0	0	0	0
*Cryptosporidium* spp.	11	11.2	11	13.3	4	7.1	4	8	1	1.8	4	7	1	2	3	4.5	39	7.6
*Cyclospora* spp.	14	14.3	7	8.4	5	8.9	0	0	1	1.8	3	5.3	0	0	1	1.5	31	6
*Cystoisospora* spp.	1	1	1	1.2	1	1.8	0	0	0	0	0	0	0	0	0	0	3	0.6
*Eimeria* spp.	20	20.4	23	27.7	24	42.9	10	20	1	1.8	5	8.8	17	34	12	18.2	112	21.7
*Balantidium* spp.	3	3.1	0	0	1	1.8	0	0	0	0	0	0	0	0	1	1.5	5	1
Protozoa	59	60.2	46	55.4	24	42.9	16	32	3	5.4	20	35.1	23	46	29	43.9	220	42.6
*Ascaris* spp.	0	0	0	0	0	0	0	0	0	0	0	0	0	0	0	0	0	0
Strongylida	0	0	0	0	9	16.1	15	30	5	8.9	0	0	1	2	2	3	32	6.2
Helminths	0	0	0	0	9	16.1	15	30	5	8.9	0	0	1	2	2	3	32	6.2
Total	59	60.2	46	55.4	32	57.1	31	62	7	12.5	20	35.1	24	48	31	47	250	48.4

n = number of studied; IC = Confidence interval.

Parasitic contamination in vegetables is detailed in Table 3. The highest frequency of contamination was found in red (84%) and white (82.4%) onions, followed by chili pepper (78%) ( *P*<0.0001). It is important to highlight the level of contamination detected in other vegetables that are eaten raw such as carrot (66%), radish (72.1%) and pepper (44%). When compared vegetables for the type of parasites, a higher frequency of protozoa (47.1%) than helminths (24.2%) was observed ( *P*<0.0001).

**Table 3.** Distribution of parasites according to the vegetable type analysed.

Parasites	Red onion		White onion		Potato		Carrot		Radish		Beet		Pepper		Chili pepper		Beans chocho		Total	
	n=50	%	n=51	%	n=52	%	n=53	%	n=61	%	n=51	%	n=50	%	n=50	%	n=70	%	n=488	%
*Blastocystis* sp.	28	56	16	31.4	11	21.2	12	22.6	22	36.1	9	17.6	6	12	14	28	16	22.9	134	27.5
*Entamoeba* spp.	3	6	1	2	6	11.5	6	11.3	8	13.1	13	25.5	1	2	7	14	3	4.3	48	9.8
*E. coli*	0	0	2	3.9	3	5.8	1	1.9	5	8.2	0	0.0	0	0	0	0	0	0	11	2.3
*E. hartmanni*	0	0	1	2	1	1.9	0	0	0	0	0	0.0	0	0	0	0	0	0	2	0.4
*Endolimax nana*	0	0	4	7.8	1	1.9	2	3.8	0	0	0	0.0	0	0	2	4	0	0	9	1.8
*Iodamoeba buetschlii*	0	0	0	0	0	0	1	1.9	0	0	0	0.0	1	2	0	0	0	0	2	0.4
*Giardia* spp.	8	16	6	11.8	1	1.9	3	5.7	3	4.9	2	3.9	7	14	7	14	3	4.3	40	8.2
*Chilomastix* spp.	4	8	2	3.9	1	1.9	0	0	0	0	0	0	0	0	1	2	0	0	8	1.6
*Retortamonas* spp.	0	0	0	0	0	0	1	1.9	0	0	0	0	0	0	0	0	0	0	1	0.2
*Enteromonas* spp.	0	0	0	0	0	0	0	0	0	0	0	0	0	0	0	0	0	0	0	0
*Cryptosporidium* spp.	3	6	7	13.7	2	3.8	6	11.3	3	4.9	2	3.9	0	0	2	4	5	7.1	30	6.1
*Cyclospora* spp.	2	4	2	3.9	1	1.9	5	9.4	3	4.9	6	11.8	0	0	0	0	1	1.4	20	4.1
*Cystoisospora* spp.	0	0	0	0	1	1.9	1	1.9	0	0	0	0	0	0	0	0	0	0	2	0.4
*Eimeria* spp.	10	20	10	19.6	15	28.8	13	24.5	22	36.1	17	33.3	12	24	17	34	7	10	123	25.2
*Balantidium* spp.	1	2	3	5.9	3	5.8	4	7.5	7	11.5	6	11.8	1	2	3	6	2	2.9	30	6.1
Protozoa	24	48	28	54.9	27	51.9	25	47.2	33	54.1	28	54.9	19	38	27	54	19	27.1	230	47.1
*Ascaris* spp.	0	0	0	0	0	0	0	0	0	0	2	3.9	1	2	0	0	0	0	3	0.6
Strongylida	16	32	28	54.9	22	42.3	7	13.2	15	24.6	11	21.6	1	2	15	30	0	0	115	23.6
Helminths	16	32	28	54.9	22	42.3	7	13.2	15	24.6	13	25.5	2	4	15	30	0	0	118	24.2
Total	42	84	42	82.4	40	76.9	35	66.0	44	72.1	37	72.5	22	44	39	78	30	42.9	331	67.8

n = number of studied; IC = Confidence interval.

Regarding the parasitic contamination of leafy greens, parasites were found in almost each specimen analyzed of cabbage (100%), alfalfa (90.2%) and parsley (82.4%). Cabbage had high contamination with *Eimeria* (53.8%) *(P*<0.0001) and with *Endolimax nana* (13.5%) ( *P*=0.0002), whereas lettuce was mainly contaminated with *Entamoeba* spp. (36.2%) ( *P*<0.0001), and parsley with *Blastocystis* (56.9%) ( *P*=0.0071) ( Table 4).

**Table 4.** Distribution of parasites according to the leafy green type analysed.

Parasites	Alfalfa		Lettuce		Cabbage		Chard		Parsley		Coriander		Celery		Watercress		Total	
	n=51	%	n=58	%	n=52	%	n=50	%	n=51	%	n=50	%	n=50	%	n=50	%	n=412	%
*Blastocystis* sp.	13	25.5	24	41.4	20	38.5	12	24	29	56.9	19	38	12	24	19	37.3	148	35.9
*Entamoeba* spp.	4	7.8	21	36.2	16	30.8	3	6	2	3.9	6	12	7	14	10	19.6	69	16.7
*E. coli*	0	0	1	1.7	1	1.9	0	0	1	2	0	0	0	0	0	0	3	0.7
*E. hartmanni*	0	0	0	0	0	0	0	0	0	0	0	0	1	2	0	0	1	0.2
*Endolimax nana*	5	9.8	0	0	7	13.5	0	0	2	3.9	1	2	0	0	0	0	15	3.6
*Iodamoeba buetschlii*	0	0	0	0	1	1.9	0	0	0	0	0	0	0	0	0	0	1	0.2
*Giardia* spp.	5	9.8	5	8.6	9	17.3	4	8	7	13.7	8	16	4	8	10	19.6	52	12.6
*Chilomastix* spp.	0	0	0	0	0	0	1	2	2	3.9	0	0	0	0	0	0	3	0.7
*Retortamonas* spp.	0	0	0	0	0	0	0	0	0	0	0	0	0	0	0	0	0	0
*Enteromonas* spp.	0	0	0	0	1	1.9	0	0	0	0	0	0	0	0	0	0	1	0.2
*Cryptosporidium* spp.	1	2	3	5.2	2	3.8	1	2	5	9.8	3	6	4	8	5	9.8	24	5.8
*Cyclospora* spp.	0	0	1	1.7	4	7.7	0	0	1	2	2	4	4	8	0	0	12	2.9
*Cystoisospora* spp.	0	0	0	0	1	1.9	1	2	0	0	0	0	0	0	0	0	2	0.5
*Eimeria* spp.	26	51	17	29.3	28	53.8	14	28	13	25.5	9	18	17	34	14	27.5	138	33.5
*Balantidium* spp.	7	13.7	8	13.8	11	21.2	5	10	5	9.8	8	16	9	18	9	17.6	62	15
Protozoa	33	64.7	37	63.8	45	86.5	23	46	33	64.7	25	50	30	60	27	52.9	253	61.4
*Ascaris* spp.	1	2	0	0	0	0	0	0	0	0	0	0	1	2	0	0	2	0.5
Strongylida	13	25.5	11	19	11	21.2	6	12	8	15.7	6	12	9	18	6	12.0	70	17
Helminths	13	25.5	11	19	11	21.2	6	12	8	15.7	6	12	9	18	6	12.0	70	17
Total	46	90.2	43	74.1	52	100	30	60	42	82.4	31	62	39	78	34	66.7	317	76.9

n = number of studied; IC = Confidence interval.

The comparative analysis of parasitic contamination rates ( Table 5) showed higher parasites percentages in vegetables + leafy greens: total (72%) ( *P*<0.0001), protozoa (53.7%) ( *P*<0.0001) and helminths (20.9%) ( *P*<0.0001). A higher prevalence of *Eimeria* (29%) *(P*=0.0027), *Entamoeba* spp. (13%) ( *P*<0.0001), *Giardia* (10.2%) ( *P*=0.0007), and *Balantidium* (10.2%) ( *P*<0.0001) was found respect to the fruits. In contrast, higher percentages of *Blastocystis* (37.4%) ( *P*=0.0199) and *Cyclospora* (6%) ( *P*=0.0313) were found in fruits respect to vegetables.

**Table 5.** Comparation of parasitic contamination between fruits, vegetables and leafy greens.

Parasites	Fruit			Vegetables + Greens leafy		
	n=516	%	IC	n=900	%	IC
*Blastocystis* sp.	193	37.4	(33.2-41.6)	282	31.3	(28.3-34.3)
*Entamoeba* spp.	29	5.6	(3.6-7.6)	117	13	(10.8-15.2)
*E. coli*	9	1.7	(0.6-2.9)	14	1.6	(0.8-2.4)
*E. hartmanni*	2	0.4	(0-0.9)	3	0.3	(0-0.7)
*Endolimax nana*	31	6	(4.0-8.1)	24	2.7	(1.6-3.8)
*Iodamoeba buetschlii*	2	0.4	(0-0.9)	3	0.3	(0-0.7)
*Giardia* spp.	26	5	(3.2-7.0)	92	10.2	(8.2-12.2)
*Chilomastix* spp.	6	1.2	(0.3-2.1)	11	1.2	(0.5-1.9)
*Retortamonas* spp.	0	0	0	1	0.1	(0-0.3)
*Enteromonas* spp.	3	0.6	(0-1.3)	1	0.1	(0-0.3)
*Cryptosporidium* spp.	39	7.6	(5.3-9.9)	54	6	(4.4-7.6)
*Cyclospora* spp.	31	6	(4.0-8.1)	32	3.6	(2.4-4.8)
*Cystoisospora* spp.	3	0.6	(0-1.2)	4	0.4	(0-.80)
*Eimeria* spp.	112	21.7	(18.1-25.3)	261	29	(26.0-32.0)
*Balantidium* spp.	5	1	(0.1-1.8)	92	10.2	(8.2-12.2)
Protozoa	220	42.6	(38.4-46.9)	483	53.7	(50.4-57.0)
*Ascaris* spp.	0	0	0	5	0.6	(0.1-1.1)
Strongylida	32	6.2	(4.1-8.3)	185	20.6	(18.2-23.6)
Helminths	32	6.2	(4.1-8.3)	188	20.9	(18.2-23.6)
Total	250	48.4	(44.1-52.7)	648	72	(69.1-74.9)

n = number of studied; IC = Confidence interval.

Finally, when parasitic contamination was compared between leafy greens (76.9%) and vegetables (67.8%), a statistically significant difference was found ( *P* =0.0024) (see Table 6), including the highest contamination of leafy greens with *Blastocystis* (35.9%) ( *P*=0.0064), *Eimeria* (33.5%) ( *P*=0.0063), *Balantidium* (15.1%) ( *P*<0.0001), *Entamoeba* spp. (16.8%) ( *P*=0.0021) and *Giardia* (12.6%) ( *P*=0.0290). In contrast, vegetables were found to be more contaminated by helminths than leafy greens (24.2%) ( *P*=0.0082), mainly represented by Strongylida (23.6%) ( *P*=0.0150).

**Table 6.** Comparation of parasitic contamination between vegetables and leafy greens.

Parasites	Vegetables			Leafy Greens		
	Total			Total		
	n=488	%	IC	n=412	%	IC
*Blastocystis* sp.	134	27.5	(23.5-31.4)	148	35.9	(31.3-40.6)
*Entamoeba* spp.	48	9.8	(7.2-12.5)	69	16.8	(13.1-20.4)
*E. coli*	2	0.4	(0-1)	1	0.2	(0-0.7)
*E. hartmanni*	11	2.3	(0.9-3.6)	3	0.7	(0-1.6)
*Endolimax nana*	2	0.4	(0-1)	1	0.2	(0-0.7)
*Iodamoeba buetschlii*	9	1.8	(0.7-3.0	15	3.6	(1.8-5.5)
*Giardia* spp.	40	8.2	(5.8-10.6)	52	12.6	(9.4-15.8)
*Chilomastix* spp.	8	1.6	(0.5-2.8)	3	0.7	(0-1.6)
*Retortamonas* spp.	1	0.2	(0-0.6)	0	0	(0-0)
*Enteromonas* spp.	0	0	(0-0)	1	0.2	(0-0.7)
*Cryptosporidium* spp.	30	6.2	(4.0-8.3)	24	5.8	(3.6-8.1)
*Cyclospora* spp.	20	4.1	(2.3-5.9)	12	2.9	(1.3-4.5)
*Cystoisospora* spp.	2	0.4	(0-1)	2	0.5	(0-1.2)
*Eimeria* spp.	123	25.2	(21.4-29.1)	138	33.5	(28.9-38.1)
*Balantidium* spp.	30	6.2	(4.0-8.3)	62	15.1	(11.6-18.5)
Protozoa	230	47.1	(42.7-51.6)	253	61.4	(56.7-66.1)
*Ascaris* spp.	3	0.6	(0-1.3)	2	0.5	(0-1.2)
Strongylida	115	23.6	(20.4-28)	70	17	(13.4-20.6)
Helminths	118	24.2	(20.4-28)	70	17	(13.4-20.6)
Total	331	67.8	(63.7-72)	317	76.9	(72.8-81.0)

n = number of studied; IC = Confidence interval.

## Discussion

This study uncovers significant parasitic contamination in fruits (48.4%), vegetables (67.8%), and leafy greens (76.9%), from San Andrés a principal agricultural hub in the Ecuadorian Andes, attributed to poor hygiene practices in agriculture. The detection of multiple enteric parasites in these foods highlight the potential risk of transmitting infections if consumed without adequate sanitation. The local, national and international distribution of these foods, amplifies the risk of disseminating parasites to non-endemic regions, thereby increasing the likelihood of disease outbreaks as it was shown in studies on leafy greens and berries ( Tefera et al., 2018; Marques et al., 2020; Barlaam et al., 2021, 2022; Faria et al., 2023).

Direct contact with human and animal excrements is a potential source of contamination of anthroponotic and zoonotic parasites for vegetables. It is also possible that free-living parasites (Strongylida) contaminate the crop products, being considered an insignificant finding in comparison with parasite prevalence’s reaching 97.3%. in humans ( González et al., 2022) and 90.3% in animals ( González et al., 2021).

When comparing the results of vegetable contamination from the San Andres capital, with an overall prevalence of 63.4%, (fruits 48.4% and vegetables 67.8%), was lower than the detected in provinces located at high altitudes and more indigenous populated with overall prevalence of 70.6% (fruits 67.1% and vegetables 73.6%) ( González-Ramírez et al., 2022). Urban area used to have access to better methods of sanitation, cleaner restrooms with proper septic tanks, drinking water, and overall, more preventive education and information on food handling than rural areas. This could explain why central town of San Andres showed lower percent of parasitic contamination in their vegetable products when compared to the contamination rate determined in products from rural provinces located at high altitudes (63.4% *vs* 70.6%) ( González-Ramírez et al., 2022).

In the present study, leafy greens were more contaminated (76.9%) than vegetables and fruits, probably since these maintain contact with the soil and organic fertilizers from the beginning as seedlings until they are fully grown, and external leaves allow protection for internal plant parts in contact with contaminated soil. The greater parasitic contamination of leafy greens has been explained by the irregularities of their leaves and the roughness of their surface that allows the adhesion of infectious parasitic forms that persist in the environment ( Tefera et al., 2018; Temesgen et al., 2022; Falcone et al., 2023).

Vegetables were the second most contaminated products after leafy greens, surpassing fruits, which is explained by the greater contact they maintain with the soil. The rooted vegetables (tubercle) were found to be highly parasitized by nematodes (24.3%), possibly because they grew under the ground. Noteworthy, onions (54.9%), carrots (13.2%) and radishes (24.6%) are frequently consumed raw and can function as efficient vehicles for parasites. Evidence of these tubers exhibit significant rates of parasitic contamination has been previously reported elsewhere ( Puig-Peña et al. 2013; Yahia et al. 2023).

Fruits growing on trees or bushes were found less contaminated than creeping fruits. It is possible that these fruits have been in direct contact with the irrigation water ( Esteban et al., 2002; González-Ramírez et al., 2020), organic fertilizers and the soil ( Dixon, 2016; Barlaam 2021, 2022; Falcone et al., 2023). However, the roughness of its surface is also a condition that can also influence the contamination of blackberry and peach ( Tefera et al., 2018), the texture of its surface allows the adhesion of parasites dispersed by wind, insects or farmers’ hands ( Dixon, 2016; Machado-Moreira et al. 2019).

Animal feces are a nutrient-rich fertilizer for agricultural systems and offer a low-cost solution ( Daniels et al., 2016). However, without prior treatment (composting, storage, chemical treatment, drying, fermentation), it is a vehicle for microorganisms ( Amissah-Reynolds et al., 2020). This risk factor was identified in the agricultural practice of San Andrés. ( González-Ramírez et al., 2021), suboptimal crop management practices, including open defecation near crops without handwashing by farmers due to a lack of portable toilets, irrigation of crops with contaminated water and persistent unsatisfactory sanitary conditions in the areas where they sell their products ( González-Ramírez et al., 2021, 2022).

Contaminated water from canals and wells ( Esteban et al., 2002; González-Ramírez et al., 2020), spreads parasites and carries a high health risk, when is utilized for crop irrigation, supply animals, dilution of fertilizers and fungicides, washing machinery, equipment, and utensils work ( Dixon 2016). Rain and sprinkler irrigation transport microorganisms from soil to plants when drops splash ( Efstratiou et al., 2017). Besides, the wind lifts particles of dust from the ground that aid adherence of parasitic to the vegetables of trees or shrubs ( Machado-Moreira et al., 2019), which explains the finding of Strongylida on the woolly surface of peaches.

Insects and animals’ action, must be considered ( González-Ramírez et al., 2021, 2022). However, the greatest influence is exerted by the agricultural activities carried out by farmers, when handle vegetables without hygienic measures, during planting, harvesting, transporting, storage, and washing ( Dixon, 2016; Machado-Moreira et al., 2019). Parasitic contamination of vegetables harvested in this area could be one of the causes of the high prevalence in humans (98.2%), without ruling out the action of water contamination (57-100%), mechanical vectors (52.7%), and animals (90.3%) in these communities ( González-Ramírez et al., 2020, 2021, 2022).

After evaluating the crop contamination in the area, we warn about the need to sanitize the products before consuming them raw, because the contamination detected in this Andean area can also occur in other countries where producers do not apply hygienic measures ( Falcone et al., 2023). These results suggest the need to integrate parasites to the list of contaminants that are managed in the microbiological criteria required by the Ecuadorian Technical Standard ( INEN, 2016). Monitoring only Escherichia coli in vegetables is not a good indicator to guarantee food safety, due low infectious doses of parasites constitute a risk ( Barlaam et al., 2022).

It is advisable to consider the potential effects of productive activities on food security; these include identifying and minimizing contamination of soil, water, or any other agent used in production, and monitoring animal health so that it does not represent threats ( Tefera et al., 2018). Authorities must develop mitigation plans that involve hygiene education programs for producers and consumers. In addition, facilitate the implementation of more advanced technological procedures to improve the diagnosis of microorganisms in laboratories, as well as field routines to improve the quality and safety of these foods in accordance with standards ( Temesgen et al., 2022).

In developing countries, where molecular analyzes cannot be done, due to their high cost and the difficulty in permission to transport samples to a molecular laboratory. The sedimentation technique, staining, and micrometric measurement allow the identification of parasites at low cost (it being essential that analysts are trained). We are aware of the importance of determining parasitic species by molecular methods, for epidemiological control and we recognize, the limitation of the present study in which the analyzes were carried out by microscopic diagnosis, although insufficient for specific identification, was relevant due to the percentage of parasite genera detected.

The prevalence of our study (63.4%) has been one of the highest, when compared to those described in Ethiopia 54.4% (Bekele et al., 2017); Brazil 50.9% ( Luz et al. 2017); Ghana 57.5% ( Kudah et al., 2018); Thailand 35.1% ( Punsawad et al., 2019); Syria 34.4% ( Al Nahhas and Aboualchamat, 2020); Peru 45.3% ( Lucas et al., 2023); and Argentina 58.6% ( Falcone et al., 2023). The parasite detected are similar to those reported in Andean area of Peru ( Pérez-Cordón et al.,2008), with a greater number of protozoa than helminths.

Our results differ from those obtained in Brazil ( Honório Santos et al., 2019), with prevalence of 70% in fruits: guava (90%), lemon and apple (70%) and grape (50%). The highest prevalence in this study was of the helminths *A. lumbricoides*, Ancylostomids, *Taenia* spp., and *E. vermicularis*, followed by *B. coli* and *E. coli.* These differences might be due to the high altitude of San Andrés (3,020–6,310 m above sea) could affect the evolution of soil-transmitted helminths due to the extreme environmental conditions such as low temperatures (0–19 °C), intense solar radiation and low rainfall (250 and 500 mm/year). In addition, these conditions affect the soil composition which is constituted by very thin layers of lithic materials of volcanic origin ( González-Ramírez et al., 2022). Effect of altitude on helminths has been reported elsewhere ( Chammartin et al., 2013).

Interestingly, in San Andrés, there were significant differences between contamination in leafy green types, which is consistent with the results that indicate highest-contaminated in lettuce, reaching rates of 83% Bolivia ( Rodríguez et al., 2015); 54.2% Ghana ( Kudah et al., 2018); 29.5% Syria ( Al Nahhas and Aboualchamat, 2020); 80% Brazil ( De Farias et al., 2021); 82.3% Ecuador ( Bracho-Mora et al., 2022); 64.7% Argentina ( Falcone et al., 2023); 23.8% Portugal ( Faria et al., 2023).

Food-borne transmission of parasites is an emerging issue in countries around the world, although, verifying the transmission of parasites through food is not easy, there is a report from the Center for Science in the Public Interest in the United States, which found that, between 2004 and 2013, the consumption of fresh produce was associated with a total of 193,754 illnesses across 9,626 outbreaks. Of the total number of reported outbreaks, the U.S. Centers for Disease Control and Prevention were able to identify both the food source and the contaminant in fewer than 40 percent ( CSPI, 2015).

Warning about contamination of unpasteurised apple juice, onions, salads, lettuce, basil, sandwiches, fruit salads, and raspberries with *Giardia*, *Cryptosporidium*, and *Cyclospora* ( Dixon, 2016). Outbreaks, associated with the consumption of berries, specifically noting *Cyclospora* and *Trypanosoma*, this latter one associated with the consumption of açaí berries or their beverages ( Tefera et al., 2018). Barlaam et al. (2021, 2022) confirm the contamination of produces exported from endemic to non-endemic countries by detecting *C. cayetanensis*, *E. histolytica*, and *Cryptosporidium* in berries imported to Italy from Peru, indicating a serious risk from contaminated produce.

Detection of foodborne parasites in produced has been reported in Latin American countries using the spontaneous sedimentation technique and optical microscopy. Contamination rates include: 77.78% vegetables in Venezuela ( Cazorla-Perfetti et al., 2013), 83% lettuces in Bolivia ( Rodríguez et al., 2015), 100% lettuces in Colombia ( Polo et al., 2016), 50.9% vegetables in Brazil ( Luz et al., 2017), 56.7% vegetables in Peru ( Benites Salcedo et al., 2019), 82.3% lettuces in Ecuador ( Bracho-Mora et al., 2022), 70.6% fruits-vegetables in Ecuador ( González-Ramírez et al., 2022), 45.3% lettuces in Peru ( Lucas et al., 2023), and 58.6% leafy vegetables in Argentina ( Falcone et al., 2023).

In Europe, studies using molecular techniques have reported lower prevalences of parasites in fresh produce compared to Latin America. In Italia, Caradonna et al. (2017) detected *G. duodenalis* (0.6%), *T. gondii* (0.8%), *Cryptosporidium* spp. (0.9%), *C. cayetanensis* (1.3%), *B. hominis* (0.5%), and *D. fragilis* (0.2%) to overall contamination of 4.2% in salads. Barlaam et al. (2021, 2022) identified *G. duodenalis* (4.6%), *Entamoeba histolytica* (1%), and *Cryptosporidium* spp. (5.1%) in berries and salad. *E. multilocularis* (1.39%) in salad. Temesgen et al. (2022) identified *T. gondii* (2.9%), *C. cayetanensis* (6.6%), and *Cryptosporidium* spp. (8.3%) in berries.

On the contrary, in Spain, Trelis et al. (2022) demonstrated higher levels of contamination in green leafy vegetables, with *G. duodenalis* (23.3%) and *Cryptosporidium* spp. (7.8%), marketed in the city of Valencia. In the same city, Moreno-Mesonero et al. (2023) identified a greater variety of species than Trelis et al., in leafy greens and strawberries: *Acanthamoeba* (65.5%), *T. gondii* (37.2%), *Vermamoeba vermiformis* (17.3%), *C. cayetanensis* (12.7%), *Cryptosporidium* spp. (6.8%), *Blastocystis* sp. (1.8%), and *Giardia* sp. (1.7%). Similarly, in Portugal, Marques et al. (2020) have documented a contamination rate of 40% in fruits and vegetables with *T. gondii.*

When comparing findings from agricultural products from industrialized nations with our study in Ecuador, we obtained a higher prevalence and diversity of human and veterinary parasitic species. For example, in Italy ( Barlaam et al. 2021, 2022), Spain ( Trelis et al., 2022); Portugal ( Marques et al., 2020), Norway ( Temesgen et al., 2022) and Sweden ( Ahlinder et al., 2022), coccidia were mainly identified. Notably, species prioritized in Europe such as *Echinococcus*, *T. gondii*, *Toxocara,* and *Fasciola* were not detected in our research ( Bouwknegt et al., 2018). However, European studies used to be done on fruits and vegetables from supermarkets, which are pre-washed or disinfected prior to sale, in contrast to our agricultural products directly obtained from farmers’ fields. This is a factor that likely influences the observed prevalence rates.

The place sampling was identified as critical nodes for contamination ( Lucas et al., 2023), this is linked to suboptimal crop management practices, including open defecation, the absence of handwashing due to a lack of portable toilets in the fields; and the use of fresh animal excrement as fertilizer ( Amissah-Reynolds et al., 2020). Farmers neglect to sanitize work tools like shovels, picks, rakes, and wheelbarrows, facilitating the transfer of parasites between different crops. Furthermore, unsatisfactory sanitary conditions persist in the areas where they sell their products ( González-Ramírez et al., 2022).

In Latin America there are the highest records of contamination in vegetables ( Cazorla-Perfetti et al., 2013; Rodríguez et al., 2015; Polo et al., 2016; Luz et al., 2017; Benites Salcedo et al., 2019; Bracho-Mora et al., 2022; González-Ramírez et al., 2022; Lucas et al., 2023; Falcone et al., 2023), being countries endemic for parasites, from there they spread to other countries nonendemic, through fresh vegetables. Developing countries have not been able to control their parasites due to low socioeconomic and hygienic levels, and the inability to offer adequate health and education infrastructure that can change people’s habits and prevent environmental pollution.

The implementation of control measures in fresh produce preharvest and postharvest, as well as an adequate sanitary hygienic level of the producer, handler, and consumer, will be crucial to minimize the food transmission of protozoa and helminths. To control parasites at the time of cultivation and harvest, irrigation with properly treated water, monitoring the health and hygiene of agricultural workers, improving agricultural sanitation, and restricting access of livestock and other animals to crops and surface water bodies (building adequate drinking troughs) are needed. Additionally, proper construction and maintenance of septic tanks is important to prevent contamination by overflow ( Tefera et al., 2018).

Unsafe agricultural practices are used very commonly by small farmers mainly in developing countries. To mitigate this problem, it is necessary to use treated water for irrigation, washing fresh produce, washing hands, and equipment. Good hygienic practices by farm workers involved in the cultivation, harvesting, and handling of produce are another important means of reducing the likelihood of contamination in endemic regions ( Trelis et al., 2022), to ensure the safety of products from Latin American countries, and are not excluded from international markets, when implementing import restrictions from endemic countries, as suggested ( Barlaam et al., 2021).

The recommendation is to impart hygienic practices through health education targeting farmers, traders, and consumers ( Tefera et al., 2018; Trelis et al., 2022; Falcone et al., 2023). If programs are executed to guarantee sanitary control in the farms and the objectives of food security are achieved in production, exports would increase, translating to an increase in the economic income of the producing countries.

## Conclusion

This study has highlighted significant parasitic contamination (63.4%), so much in fruits (48.4%), vegetables (67.8%), and leafy greens (76.9%), underscoring the potential health risks associated with the consumption of these products in their raw form without adequate hygiene practices. It illustrates how such products can become vehicles for the transmission of enteroparasites to both humans and animals, regardless of whether the area is endemic or non-endemic, where these items are distributed. Consequently, this research underscores the imperative for stringent hygienic protocols in the cultivation and harvesting phases. Effective mitigation strategies include the treatment of soil, manure, and irrigation water utilised in the agricultural process, alongside the enforcement of thorough disinfection practices prior to consumption.

## Data availability

### Underlying data

Figshare: Parasitic contamination of fruits, vegetables and leafy greens harvested in an Andean agricultural area, https://doi.org/10.6084/m9.figshare.22313335.v2 ( González-Ramírez *et al*., 2023).

This project contains the following underlying data:

• Data parasites fruits vegetables Ecuador.xlsx

Data are available under the terms of the Creative Commons Zero “No rights reserved” data waiver (CC0 1.0 Public domain dedication).
