# Supplementary material for: Field study of parasitic contamination of fruits, vegetables and leafy greens in the Ecuadorian Andes
Source: F1000Res. 2024 Mar 12;12:532. Originally published 2023 May 23. [Version 2] doi: 10.12688/f1000research.132957.2 (PMC10808852; doi:10.12688/f1000research.132957.2)
Supplement: Peer review history

# Reviewer report: Reviewer response for version 2

**DOI:** 10.5256/f1000research.163126.r254884

**Published:** 2024-04-13

**Authors:** Zulbey Rivero

**Affiliations:** 1 Chair of Parasitology, Universidad Tecnica de Manabi, Portoviejo, Manabí Province, Ecuador

I agree with the corrections made.

Is the work clearly and accurately presented and does it cite the current literature?

Yes

If applicable, is the statistical analysis and its interpretation appropriate?

Yes

Are all the source data underlying the results available to ensure full reproducibility?

Yes

Is the study design appropriate and is the work technically sound?

Yes

Are the conclusions drawn adequately supported by the results?

Yes

Are sufficient details of methods and analysis provided to allow replication by others?

Yes

Reviewer Expertise:

NA

I confirm that I have read this submission and believe that I have an appropriate level of expertise to confirm that it is of an acceptable scientific standard.

# Reviewer report: Reviewer response for version 2

**DOI:** 10.5256/f1000research.163126.r254887

**Published:** 2024-03-12

**Authors:** Alessandra Barlaam

**Affiliations:** 1 Department of Agriculture, Food, Natural Resources and Engineering (DAFNE), University of Foggia, Foggia, Italy

The article has been significantly improved, therefore, in my opinion it can be accepted for approved in the current form.

Is the work clearly and accurately presented and does it cite the current literature?

Partly

If applicable, is the statistical analysis and its interpretation appropriate?

Yes

Are all the source data underlying the results available to ensure full reproducibility?

Partly

Is the study design appropriate and is the work technically sound?

Partly

Are the conclusions drawn adequately supported by the results?

Partly

Are sufficient details of methods and analysis provided to allow replication by others?

Partly

Reviewer Expertise:

Parasitology; parasitic diseases; zoonoses; foodborne parasites; food safety;

I confirm that I have read this submission and believe that I have an appropriate level of expertise to confirm that it is of an acceptable scientific standard.

# Reviewer report: Reviewer response for version 1

**DOI:** 10.5256/f1000research.145917.r228641

**Published:** 2024-01-30

**Authors:** Zulbey Rivero

**Affiliations:** 1 Chair of Parasitology, Universidad Tecnica de Manabi, Portoviejo, Manabí Province, Ecuador

The article meets the objective proposed from the beginning and the introduction is brief but clear enough. The methodology is well described, but there is something that needs to be clarified and about which I am asking the authors in the comments placed as stickers in the PDF file. The most important findings are highlighted in the results, however it would be interesting if the authors could report which morphotypes of Blastocystis sp were detected in the samples evaluated. In addition, they must indicate if samples were found that showed contamination by more than one parasitic species in the vegetable or fruit analyzed. The discussion is fine, but depending on what you answer regarding polyparasitism and Blastocystis, you may have to add some of this to the discussion.

Is the work clearly and accurately presented and does it cite the current literature?

Yes

If applicable, is the statistical analysis and its interpretation appropriate?

Yes

Are all the source data underlying the results available to ensure full reproducibility?

Yes

Is the study design appropriate and is the work technically sound?

Yes

Are the conclusions drawn adequately supported by the results?

Yes

Are sufficient details of methods and analysis provided to allow replication by others?

Yes

Reviewer Expertise:

Parasitology, Diagnostic Techniques

I confirm that I have read this submission and believe that I have an appropriate level of expertise to confirm that it is of an acceptable scientific standard, however I have significant reservations, as outlined above.

# Response

**Published:** 2024-02-25

**Authors:** LUISA CAROLINA GONZALEZ

**Affiliations:** Laboratorio Clínico, Universidad Nacional de Chimborazo, Riobamba, Chimborazo Province, Ecuador

**Revisor 4**

 We thank the reviewer for the importance has given to our research, the time spent correcting the manuscript, their knowledge, experience, and willingness to improve the article.

**Comment:**

 The article meets the objective proposed from the beginning and the introduction is brief but clear enough. The methodology is well described, but there is something that needs to be clarified and about which I am asking the authors in the comments placed as stickers in the PDF file. The most important findings are highlighted in the results; however it would be interesting if the authors could report which morphotypes of *Blastocystis* sp were detected in the samples evaluated. In addition, they must indicate if samples were found that showed contamination by more than one parasitic species in the vegetable or fruit analyzed. The discussion is fine, but depending on what you answer regarding polyparasitism and *Blastocystis*, you may have to add some of this to the discussion.

**Response**

 Observations made in the text

**Investigation design**

 A field study, cross-sectional, observational and descriptive, was carried out during 1 month of rain / 7 months of drought. The snowball sampling technique was applied, whereby a grower helped locate the nearest farm and so on. All types of products found were included in the sampling (1,416 samples in total); the inclusion criteria were that all agricultural products must come from San Andrés fields and those not cultivated in the community were excluded.

**A separatory funnel was not used; it has been a translation error**

 The reviewer is right. We apologize for the writing mistake. The allusion to a funnel has been deleted (see line 6 paragraph 8)

**It would be interesting if the results indicated whether, in some samples, more than one parasitic species was obtained from the studied vegetables, lettuce, or fruit. Something akin to polyparasitism in humans.**

 This is a very important observation of the reviewer as most samples already presented polyparasitism. We have now described this in our results as well as in the discussion section (please see lines ???? in the new version of the manuscript).

**Given that *Blastocystis* was the primary microorganism found, if the authors have the information, it would be important to indicate which morphotype was most frequently observed.**

 The morphotype of *Blastocystis *identified in the samples is now indicated in the result section (please, see lines 9 and 10)

**I assume that leafy vegetables were combined to enable pairwise comparisons. If that is the case, it should be noted that, for statistical reasons, sample groups were combined**

 The reviewer is right. We have now indicate that some groups were combined for statistical purpose.

# Reviewer report: Reviewer response for version 1

**DOI:** 10.5256/f1000research.145917.r228648

**Published:** 2024-01-24

**Authors:** Alessandra Barlaam

**Affiliations:** 1 Department of Agriculture, Food, Natural Resources and Engineering (DAFNE), University of Foggia, Foggia, Italy

The aim of the study was to assess the occurrence of the parasitic contamination of fruits, vegetables and leafy greens grown in Ecuador, one of the most important exporters of fresh vegetables. In total 63.4% of the samples were found positive for a variety of parasites which highlights the need to improve and look into the management of the products from farm to fork.

**Abstract:**

 In the Abstract, Methods: “Each sample were washed” should be replaced with “Each sample was washed”.

 Abstract, Results: The English language should be revised as the paragraph is not as clear as it should be. In addition, some repetitions occur (the most contaminated) and the use of , and ; is confusing.

 Abstract, Conclusion: Delete “From these crops” given that it is a general concept regardless of the obtained results.

**Introduction:**

 Given the variety of fresh produce included in the study and the number of parasites detected, the introduction is too concise and should be enriched. In addition, the importance of leafy greens as vehicles of foodborne parasites is emphasized whereas the role vegetables and fruits, including berries (also analyzed in this study and for which relevant and recent publications are available) is completely overlooked.

 When discussing import of fresh produce from endemic countries and the spread of parasites to nonendemic ones the authors fail to include relevant and recent bibliography on the subject. There are, in fact, recent studies that show how fresh produce imported from countries endemic for certain parasites have been found contaminated in the importing country. These articles have been completely overlooked and should be included. I recommend:

- Barlaam *et al.* (2021 ^1^);
- Temesgen *et al. *(2022 ^2^);
- Barlaam *et al.* (2022 ^3^).

**Methods:**

 The data are quite old (the samples were collected four years ago) and so is the methodology used for processing the fresh produce. The detection is also not quite in step with the times. Specifically, in the present study, the occurrence of parasites in fresh produce was investigated by microscopy although it is not clear how the genera and species were verified. Nowadays, with molecular tools available it would be auspicable to use them not only to verify the microscopy results but also to gather more information on the detected parasites (pathogenicity, zoonotic relevance etc.). I would like to ask the authors whether the samples were not tested molecularly because of lack of equipment/resources or for different reasons.

**Tables:**

 In all the tables there is a line that refers to “Protozoa”. I don’t understand why given that the protozoa identified are listed individually. Please clarify.

 Abbraviations should be written in full at the bottom of the table (IC with a * that links the abbreviation and the extended form).

**Discussions:**

 The discussions are well organized and the key concepts regarding parasitic contamination of fresh produce are covered.  

 First paragraph: See comment in the Introduction section about the risk that these products represent for people living in non-endemic areas. Please cite relevant literature.

 When discussing contaminated berry products and the surface of such products is discussed, the following paper should be taken into consideration: Tefera *et al.* (2018 ^4^).

 The paragraph starting with “Food-borne transmission of protozoan parasites…” needs to be amended in order to clarify two different concepts: foodborne transmission of protozoan parasites and detection of foodborne parasites into fresh produce. When listing the cases in which parasitic contamination of fresh produce occurred, the authors do not use the most recent bibliography available. They include, in fact, older articles, but they overlook more recent publications on the subject (among others, Barlaam *et al.*, 2021; Barlaam *et al.*, 2022; Temesgen *et al.*, 2022; Marques *et al.* (2020 ^5^); Faria *et al. *(2023 ^6^). Please update.

**Conclusion:**

 It may start from three paragraph above (“In these tropical countries,..”) since they are very general concluding paragraphs.

**References:**

 In some parts of the manuscript as stated in the previous comments the references are rather dated. For many subjects the authors write about, in fact, they cite articles that are not among the most relevant and recent on the subject. I recommend doing another bibliographic research and going through the references again.

**English:**

 The use of the English language is generally good, however, some misspellings, inaccuracies and errors in the sentence structure have been spotted throughout the text and a further revision is highly recommended.

 Taking everything into account this study has some limitations and the manuscript has some flaws, however, these data shed light on an important matter which is parasitic contamination of fresh produce in developing Countries which play a key role in our economy as exporters. This means that such issue is not limited to the Country in topic but potentially threatening for the rest of the world. For this reason, I think that it’s important to share data as limited as they may be on the subject and raise awareness on the issue. Therefore, in my opinion the manuscript can be indexed after the points raised above are clarified and a thorough revision of the manuscript is made according to the revisions above.

Is the work clearly and accurately presented and does it cite the current literature?

Partly

If applicable, is the statistical analysis and its interpretation appropriate?

Yes

Are all the source data underlying the results available to ensure full reproducibility?

Partly

Is the study design appropriate and is the work technically sound?

Partly

Are the conclusions drawn adequately supported by the results?

Partly

Are sufficient details of methods and analysis provided to allow replication by others?

Partly

Reviewer Expertise:

Parasitology; parasitic diseases; zoonoses; foodborne parasites; food safety;

I confirm that I have read this submission and believe that I have an appropriate level of expertise to confirm that it is of an acceptable scientific standard, however I have significant reservations, as outlined above.

## References

[1] : Contamination of fresh produce sold on the Italian market with Cyclospora cayetanensis and Echinococcus multilocularis. *Food Microbiol* .2021;98: 10.1016/j.fm.2021.103792 103792 10.1016/j.fm.2021.103792 33875219

[2] : Surveillance of berries sold on the Norwegian market for parasite contamination using molecular methods. *Food Microbiol* .2022;104: 10.1016/j.fm.2022.103980 103980 10.1016/j.fm.2022.103980 35287809

[3] : Ready-to-eat salads and berry fruits purchased in Italy contaminated by Cryptosporidium spp., Giardia duodenalis, and Entamoeba histolytica. *Int J Food Microbiol* .2022;370: 10.1016/j.ijfoodmicro.2022.109634 109634 10.1016/j.ijfoodmicro.2022.109634 35316671

[4] : Parasite contamination of berries: Risk, occurrence, and approaches for mitigation. *Food Waterborne Parasitol* .2018;10: 10.1016/j.fawpar.2018.04.002 23-38 10.1016/j.fawpar.2018.04.002 32095598 PMC7033989

[5] : Detection of Toxoplasma gondii oocysts in fresh vegetables and berry fruits. *Parasit Vectors* .2020;13(1) : 10.1186/s13071-020-04040-2 180 10.1186/s13071-020-04040-2 32268915 PMC7140358

[6] : Molecular investigation of ready-to-eat salads for Giardia duodenalis and Cryptosporidium spp. in Portugal. *Food Waterborne Parasitol* .2023;30: 10.1016/j.fawpar.2023.e00190 e00190 10.1016/j.fawpar.2023.e00190 36923349 PMC10009061

# Response

**Published:** 2024-02-25

**Authors:** LUISA CAROLINA GONZALEZ

**Affiliations:** Laboratorio Clínico, Universidad Nacional de Chimborazo, Riobamba, Chimborazo Province, Ecuador

**Revisor 3**

 We thank the reviewer for the importance has given to our research, the time spent correcting the manuscript, and their knowledge, experience, and willingness to improve the article.

**Comment 1:**

 Abstract:

 In the Abstract, Methods: “Each sample were washed” should be replaced with “Each sample was washed”.

**Response 1:**

 We have done the change

**Comment 2:**

 Abstract, Results: The English language should be revised as the paragraph is not as clear as it should be. In addition, some repetitions occur (the most contaminated) and the use of, and; is confusing.

**Response 2:**

 The English in the Abstract and Result sections has been revised and improved.  

**Comment 3:**

 Abstract, Conclusion: Delete “From these crops” given that it is a general concept regardless of the obtained results.

**Response 3:**

 The phrase has been deleted

**Comment 4:**

 Given the variety of fresh produce included in the study and the number of parasites detected, the introduction is too concise and should be enriched. In addition, the importance of leafy greens as vehicles of foodborne parasites is emphasized whereas the role vegetables and fruits, including berries (also analyzed in this study and for which relevant and recent publications are available) is completely overlooked.

**Response 4:**

 Thank you very much for these observations. The introduction has been enriched as suggested and the role of vegetables and fruits as parasitic foodborne carriers is now highlighted (see paragraphs 4, 5, and 6 in the new paper version).   

**Comment 5:**

 When discussing the import of fresh produce from endemic countries and the spread of parasites to nonendemic ones the authors fail to include relevant and recent bibliography on the subject. There are, in fact, recent studies that show how fresh produce imported from countries endemic for certain parasites have been found contaminated in the importing country. These articles have been completely overlooked and should be included. I recommend:

 •           Barlaam et al. (20211);

 •           Temesgen et al. (20222);

 •           Barlaam et al. (20223).

**Response 5:**

 They have been included as suggested (see paragraph 2 new version of paper).  

**Comment 6:**

 Methods:

 The data are quite old (the samples were collected four years ago) and so is the methodology used for processing the fresh produce. The detection is also not quite in step with the times. Specifically, in the present study, the occurrence of parasites in fresh produce was investigated by microscopy although it is not clear how the genera and species were verified. Nowadays, with molecular tools available it would be auspicable to use them not only to verify the microscopy results but also to gather more information on the detected parasites (pathogenicity, zoonotic relevance etc.). I would like to ask the authors whether the samples were not tested molecularly because of lack of equipment/resources or for different reasons.

**Response 6:**

**6.1. The data are quite old (the samples were collected four years ago)**

 The samples were collected a few years ago; however, the hygienic and sanitary practices in the area persist, and the prevalence of infection within the human populations remains constant (98.2%). No educational interventions have been implemented to enhance crop management hygiene. Additionally, indigenous communities do not readily alter their ancestral customs, suggesting that significant changes in these results are unlikely.

**6.2. The methodology used for processing the fresh produce are quite old**

 The washing and sedimentation technique used in our study (Rivero de Rodríguez et al., 1998) has been widely used, even today (Al Nahhas et al., 2020; Devera et al., 2021; Falcone et al., 2023; Lucas et al., 2023; Yahia et al., 2023; Asfaw et al., 2023; El Safadi et al., 2023). These parasitological techniques are very useful in low-resource countries, such as our case in Ecuador, since the addition of coagulant or flocculant reagents would increase the overall cost of the process.

**6.3. The detection is also not quite in step with the times**

 Optical microscopy remains the most used method in laboratories for the diagnosis of coproparasitoscopic. Its limitation has to do with the expertise of the laboratory technician to identify parasitic species. We have extensive experience in this type of diagnosis. On the other hand, we have no other diagnostic option due to the economic and logistical limitations of the area.

**6.4.** **The occurrence of parasites in fresh produce was investigated by microscopy although it is not clear how the genera and species were verified.**

 The identification of parasitic elements (eggs, larvae, cysts, oocysts) was conducted by an expert in parasitic microscopy, boasting 33 years of experience, utilizing the dimensions of the forms (using an ocular micrometer) and morphological characteristics (WHO, 2019). Given the wide variety of human, animal, and free-living parasites recoverable from plant matter, coupled with the limitations of microscopy for precise species identification, we have reported them generically (e.g., Strongylida, Ascaris spp.) without specifying species.

**6.5. Nowadays, with molecular tools available it would be auspicable to use them not only to verify the microscopy results but also to gather more information on the detected parasites (pathogenicity, zoonotic relevance etc.).**

 Undoubtedly, employing molecular methods (PCR) would yield more precise results and enable molecular epidemiology, contributing to addressing the issue of parasitic contamination. However, as previously mentioned, the application of these techniques was unfeasible in our study,  due to budget  constraints. The parasitological findings from this study have unveiled significant insights into the environmental health of crops in the Ecuadorian Andean region.

**6.6. I would like to ask the authors whether the samples were not tested molecularly because of lack of equipment/resources or for different reasons.**

 Molecular techniques were not used by lack of equipment/resources as mentioned above.

**Comment 7:**

 In all the tables there is a line that refers to “Protozoa”. I don’t understand why given that the protozoa identified are listed individually. Please clarify. Abbraviations should be written in full at the bottom of the table (IC with a * that links the abbreviation and the extended form).

**Response 7:**

 In the protozoa section of the tables, we specify the frequency and percentage of contamination for fruits, vegetables, and leafy greens individually, rather than providing a cumulative count of protozoa. The same was done with the helminth section. This approach allows us to ascertain the prevalence of each category and facilitates meaningful comparisons between the two.

**Comment 8:**

**Discussions:**

**The discussions are well organized and the key concepts regarding parasitic contamination of fresh produce are covered.**

**8.1.** **First paragraph: See comment in the Introduction section about the risk that these products represent for people living in non-endemic areas. Please cite relevant literature. When discussing contaminated berry products and the surface of such products is discussed, the following paper should be taken into consideration: Tefera et al. (2018).**

**Response 8.1:**

 Relevant literature, including the reference suggested, have been incorporated. Please, see lines 8 to Tefera et al., 2018; Marques et al., 2020; Barlaam et al., 2021, 2022; Faria et al., 2023, in the new version of the manuscript.

**8.2.** **The paragraph starting with “Food-borne transmission of protozoan parasites…” needs to be amended in order to clarify two different concepts:**

**foodborne transmission of protozoan parasites and detection of foodborne parasites into fresh produce. When listing the cases in which parasitic contamination of fresh produce occurred, the authors do not use the most recent bibliography available. They include, in fact, older articles, but they overlook more recent publications on the subject (among others, Barlaam et al., 2021; Barlaam et al., 2022; Temesgen et al., 2022; Marques et al. (2020); Faria et al. (2023). Please update.**

 Thank you for these observations.  The paragraph has been modified in order to avoid confusion with the sense in which the term is used. In addition, the references suggested has been now included (Please, paragraph see 16 new version)

**Comment 9:**

 Conclusion:

 It may start from three paragraph above (“In these tropical countries,..”) since they are very general concluding paragraphs.

**Response 9:**

 We believe that with our results, we cannot conclude what occurs in other Latin American countries.

# Reviewer report: Reviewer response for version 1

**DOI:** 10.5256/f1000research.145917.r228642

**Published:** 2024-01-24

**Authors:** Papa Kofi Amissah-Reynolds

**Affiliations:** 1 Department of Biological Sciences Education, Faculty of Science Education,College of Agriculture Education, Akenten Appiah-Menka University of Skills Training and Entrepreneurial Development, Mampong, Ghana

Generally, the manuscript is very good and relevant. However, the authors should consider the following:

1. The discussion can be revised as some aspects lack clarity and are difficult to read and/or understand.
2. The conclusion seems not to address the aim of the study. Comment on the occurrence of parasites in the conclusion.
3. Apart from using tables, can the authors consider other ways of presenting the results?

**From the discussion:**

**Paragraph 3 (When constrasting ...)**

 How does access to the urban area influence parasitic contamination?

**Paragraph 8 (Animal faeces ...)**

 Consider revising this paragraph. It is not easy to understand some aspects of this paragraph.

**Paragraph 21 (Our results ...)**

 How does altitude influence the evolution of soil-transmitted helminths? Can you provide a reference for this?

**Paragraph 23 (Food-borne ...)**

 Link the results from the developed countries you have stated to your work and explain any differences there may be.

**Paragraph 24 (Information collected ...)**

 What were the inadequate handling practices and insanitary conditions at the market?

 This article (Duedu *et al.*, 2014 ^1^) could be useful.

**Paragraph 25 (In these tropical ...)**

 Which tropical countries are you making reference to?

**Paragraphs 26 & 27**

 There are no references in these paragraphs. This article could be relevant to your work (Amissah-Reynolds *et al.*, 2020 ^2^)

Is the work clearly and accurately presented and does it cite the current literature?

Partly

If applicable, is the statistical analysis and its interpretation appropriate?

Yes

Are all the source data underlying the results available to ensure full reproducibility?

Yes

Is the study design appropriate and is the work technically sound?

Yes

Are the conclusions drawn adequately supported by the results?

Partly

Are sufficient details of methods and analysis provided to allow replication by others?

Yes

Reviewer Expertise:

Zoology, Parasitology, Zoonosis, One-Health

I confirm that I have read this submission and believe that I have an appropriate level of expertise to confirm that it is of an acceptable scientific standard.

## References

[1] : A comparative survey of the prevalence of human parasites found in fresh vegetables sold in supermarkets and open-aired markets in Accra, Ghana. *BMC Res Notes* .2014;7: 10.1186/1756-0500-7-836 836 10.1186/1756-0500-7-836 25424391 PMC4253987

[2] : Fresh Vegetables and Ready-to-eat Salads: Sources of Parasitic Zoonoses in Mampong-Ashanti, Ghana. *European Journal of Nutrition & Food Safety* .2020; 10.9734/ejnfs/2020/v12i230192 47-55 10.9734/ejnfs/2020/v12i230192

# Response

**Published:** 2024-02-25

**Authors:** LUISA CAROLINA GONZALEZ

**Affiliations:** Laboratorio Clínico, Universidad Nacional de Chimborazo, Riobamba, Chimborazo Province, Ecuador

**Revisor 2**

 We thank the reviewer for the importance has given to our research, the time spent correcting the manuscript, their knowledge, experience, and willingness to improve the article.

 Generally, the manuscript is very good and relevant. However, the authors should consider the following:

**Comment 1:**

 1. The discussion can be revised as some aspects lack clarity and are difficult to read and/or understand.

**Response 1:**

 The discussion has been revised, incorporating straightforward language and addressing all the reviewer's recommendations.

**Comment 2: The conclusion seems not to address the aim of the study. Comment on the occurrence of parasites in the conclusion.**

**Response 2:**

 A comment regarding the occurrence of parasites in the samples examined has now been included in the conclusion section, as follows:

 This study uncovers significant parasitic contamination in fruits (48.4%), vegetables (67.8%), and leafy greens (76.9%), from San Andrés a principal agricultural hub in the Ecuadorian Andes, attributed to poor hygiene practices in agriculture. The detection of multiple enteric parasites in these foods highlight the potential risk of transmitting infections if consumed without adequate sanitation. The local, national and international distribution of these foods, amplifies the risk of disseminating parasites to non-endemic regions, thereby increasing the likelihood of disease outbreaks as it was shown in studies on leafy greens and berries (Tefera et al., 2018; Marques et al., 2020; Barlaam et al., 2021, 2022; Faria et al., 2023).

 When comparing the results of vegetable contamination from the San Andres capital, with an overall prevalence of 63.4%, (fruits 48.4% and vegetables 67.8%), was lower than the detected in provinces located at high altitudes and more indigenous populated with overall prevalence of 70.6% (fruits 67.1% and vegetables 73.6%) (González-Ramírez et al., 2022), this could be explained to the access of better methods of sanitation, cleaner restrooms with proper septic tanks and overall more preventive education and information on food handling (González et al., 2022).

**Comment 3: Apart from using tables, can the authors consider other ways of presenting the results?**

**Response 3:**

 We value the recommendation to enhance the article's dynamism. While we acknowledge the appeal of diversifying presentation, we have opted for tables due to their capacity to encapsulate detailed information on parasitic contamination, which may not be effectively conveyed through figures alone.

**Comment 4: From the discussion: Paragraph 3 (When constrasting ...)**

**How does access to the urban area influence parasitic contamination?**

**Response 4:**

 Urban area used to have access to better methods of sanitation, cleaner restrooms with proper septic tanks, drinking water, and overall more preventive education and information on food handling than rural areas. This could explain why central town of San Andres showed lower percent of parasitic contamination in their vegetable products when compared to the contamination rate determined in products from rural provinces located at high altitudes   (63.4% vs 70.6%) (González-Ramírez et al., 2022).

**Comment 5: Paragraph 8 (Animal faeces ...)**

**Consider revising this paragraph. It is not easy to understand some aspects of this paragraph.**

**Response 5:**

 The paragraph was revised as suggested and now it can be read as follows:

 Animal feces are a nutrient-rich fertilizer for agricultural systems and offer a low-cost solution (Daniel et al., 2016). However, without prior treatment (composting, storage, chemical treatment, drying, fermentation), it is a vehicle for microorganisms (Amissah-Reynolds et al., 2020). This risk factor was identified in the agricultural practice of San Andrés. (González-Ramírez et al., 2021), suboptimal crop management practices, including open defecation near crops without handwashing by farmers due to a lack of portable toilets, irrigation of crops with contaminated water and persistent unsatisfactory sanitary conditions in the areas where they sell their products (González-Ramírez et al., 2021, 2022).

**Comment 6:** **Paragraph 21 (Our results ...)**

**How does altitude influence the evolution of soil-transmitted helminths? Can you provide a reference for this?**

**Response 6:**

 Our results differ from those obtained in Brazil (Honório Santos et al., 2019), with prevalence of 70% in fruits: guava (90%), lemon and apple (70%) and grape (50%). The highest prevalence in this study was of the helminths *A. lumbricoides*, Ancylostomids, *Taenia* spp., and *E. vermicularis*, followed by *B. coli* and *E. coli*. These differences might be due to the high altitude of San Andrés (3,020–6,310 m above sea) could affect the evolution of soil-transmitted helminths due to the extreme environmental conditions such as low temperatures (0–19 ºC), intense solar radiation and low rainfall (250 and 500 mm/year). In addition, these conditions affect the soil composition which is constituted by very thin layers of lithic materials of volcanic origin (González-Ramírez et al., 2022). Effect of altitude on helminths has been reported elsewhere (Chammartin et al., 2013)

**Comment 7: Paragraph 23 (Food-borne ...)**

**Link the results from the developed countries you have stated to your work and explain any differences there may be.**

**Response 7:**

 When comparing findings from agricultural products from industrialized nations with our study in Ecuador, we obtained a higher prevalence and diversity of human and veterinary parasitic species. For example, in Italy (Barlaam et al. 2021, 2022), Spain (Trelis et al., 2022); Portugal (Marques et al., 2020), Norway (Temesgen et al., 2022) and Sweden (Ahlinder et al., 2022), coccidia were mainly identified. Notably, species prioritized in Europe such as *Echinococcus,* *T. gondii*, *Toxocara*, and *Fasciola *were not detected in our research (Bouwknegt et al., 2018). However,  European studies used to be done on fruits and vegetables from supermarkets, which are pre-washed or disinfected prior to sale, in contrast to our agricultural products directly obtained from farmers' fields. This is a factor that likely influences the observed prevalence rates.

**Comment 8: Paragraph 24 (Information collected ...)**

**What were the inadequate handling practices and insanitary conditions at the market?**

**Response 8: ** The clarification has been made.

 suboptimal crop management practices, including open defecation near crops without handwashing by farmers due to a lack of portable toilets, irrigation of crops with contaminated water and persistent unsatisfactory sanitary conditions in the areas where they sell their products (González-Ramírez et al., 2021, 2022).

**Comment 9:**

**Paragraph 25 (In these tropical ...)**

**Which tropical countries are you making reference to?**

**Response 9:**

 In Latin America there are the highest records of contamination in vegetables (Cazorla-Perfetti et al., 2013; Rodríguez et al., 2015; Polo et al., 2016; Luz et al., 2017; Benites Salcedo et al., 2019; Bracho et al., 2022; González-Ramírez et al., 2022; Lucas et al., 2023; Falcone et al., 2023), being countries endemic for parasites, from there they spread to other countries nonendemic, through fresh vegetables. Developing countries have not been able to control their parasites due to low socioeconomic and hygienic levels, and the inability to offer adequate health and education infrastructure that can change people's habits and prevent environmental pollution.

**Comment 10:**

 Paragraphs 26 & 27

 There are no references in these paragraphs. This article could be relevant to your work (Amissah-Reynolds et al., 2020)

**Response 10:**

 It has been included.

 Animal feces are a nutrient-rich fertilizer for agricultural systems and offer a low-cost solution (Daniel et al., 2016). However, without prior treatment (composting, storage, chemical treatment, drying, fermentation), it is a vehicle for microorganisms (Amissah-Reynolds et al., 2020). This risk factor was identified in the agricultural practice of San Andrés. (González-Ramírez et al., 2021), suboptimal crop management practices, including open defecation near crops without handwashing by farmers due to a lack of portable toilets, irrigation of crops with contaminated water and persistent unsatisfactory sanitary conditions in the areas where they sell their products (González-Ramírez et al., 2021, 2022).

 The place sampling was identified as critical nodes for contamination (Lucas et al., 2023), this is linked to suboptimal crop management practices, including open defecation, the absence of handwashing due to a lack of portable toilets in the fields; and the use of fresh animal excrement as fertilizer (Amissah-Reynolds et al., 2020). Farmers neglect to sanitize work tools like shovels, picks, rakes, and wheelbarrows, facilitating the transfer of parasites between different crops. Furthermore, unsatisfactory sanitary conditions persist in the areas where they sell their products (González-Ramírez et al., 2022).

# Reviewer report: Reviewer response for version 1

**DOI:** 10.5256/f1000research.145917.r221529

**Published:** 2023-12-19

**Authors:** Samar Al Nahhas

**Affiliations:** 1 Department of Animal Biology, Faculty of Science, Damascus University, Damascus, Syria

This study addresses an important knowledge gap regarding the prevalence of enteric parasites on fruits, vegetables and leafy green in an agricultural area of the Ecuadorian Andes. The study also indicated the necessity of dealing with these materials, which are responsible for infection in humans and animals, by treating the soil as well as the water used in irrigation. 

  I have previously reviewed this manuscript and gave my decision to accept it.

Is the work clearly and accurately presented and does it cite the current literature?

Yes

If applicable, is the statistical analysis and its interpretation appropriate?

Yes

Are all the source data underlying the results available to ensure full reproducibility?

Yes

Is the study design appropriate and is the work technically sound?

Yes

Are the conclusions drawn adequately supported by the results?

Yes

Are sufficient details of methods and analysis provided to allow replication by others?

Yes

Reviewer Expertise:

Parasitology,  Protozoa, molecular diagnosis, infectious diseases

I confirm that I have read this submission and believe that I have an appropriate level of expertise to confirm that it is of an acceptable scientific standard.
